# Consistent patterns in 16S and 18S microbial diversity from the shells of the common and widespread red-eared slider turtle (*Trachemys scripta*)

**DOI:** 10.1371/journal.pone.0244489

**Published:** 2020-12-28

**Authors:** Matthew Parks, Cameron Kedy, Casey Skalla

**Affiliations:** Department of Biology, University of Central Oklahoma, Edmond, Oklahoma, United States of America; Carnegie Mellon University, UNITED STATES

## Abstract

Microbial communities associated with freshwater aquatic habitats and resident species are both critical to and indicative of ecosystem status and organismal health. External surfaces of turtle shells readily accumulate microbial growth and could carry representation of habitat-wide microbial diversity, since they are in regular contact with multiple elements of freshwater environments. Yet, microbial diversity residing on freshwater turtle shells is poorly understood. We applied 16S and 18S metabarcoding to characterize microbiota associated with external shell surfaces of 20 red-eared slider (*Trachemys scripta*) turtles collected from varied habitats in central and western Oklahoma, and ranging to southeast Iowa. Shell-associated microbial communities were highly diverse, with samples dominated by Bacteroidia and alpha-/gamma-proteobacteria, and ciliophoran alveolates. Alpha diversity was lower on turtle shells compared to shallow-water-associated environmental samples, likely resulting from basking-drying behavior and seasonal scute shedding, while alpha diversity was higher on carapace than plastron surfaces. Beta diversity of turtle shells was similarly differentiated from environmental samples, although sampling site was consistently a significant factor. Deinococcus-Thermus bacteria and ciliophoran alveolates were recovered with significantly higher abundance on turtle shells versus environmental samples, while bacterial taxa known to include human-pathogenic species were variably more abundant between shell and environmental samples. Microbial communities from a single, shared-site collection of the ecologically similar river cooter (*P*. *concinna*) largely overlapped with those of *T*. *scripta*. These data add to a foundation for further characterization of turtle shell microbial communities across species and habitats, with implications for freshwater habitat assessment, microbial ecology and wildlife conservation efforts.

## Introduction

A number of cultural traditions hold that the world rests on the back of a giant turtle (i.e., the ‘world turtle’) [1]. This folkloric concept is also intriguing at the microbial scale, as the turtle shell provides a unique and living substrate for microbial colonization, ecology and evolution–potentially a world of microbial diversity. The turtle shell microbiome may simultaneously serve as an indicator of both turtle health and overall ecosystem status. Very generally, the external microbiomes of organisms are influenced by climatic effects, rather than “top-down” or species-specific effects [2]. This suggests that turtles could be susceptible to pathogenic microbial growth on external surfaces, including for novel reptile-associated [3] and known human pathogens [4] present in the environment. Freshwater turtles are also known to utilize a diversity of areas in shallow to medium-depth aquatic habitats, dependent on interactions between and within turtle species, abiotic factors, and disturbance [5–10]. The exceptional capacity of many freshwater turtles to withstand anoxic conditions also allows them to exploit shallow photic to deeper aphotic benthic habitats [11]. Since the turtle shell is in direct contact with a wide range of local environmental microbial communities, it may represent an important microbial accumulator and could thus serve as a proxy for monitoring and predicting habitat quality.

Currently, very little is known about microbial communities residing on turtle shells, although this is an area of recent active research. Epibiont colonization and community structure on turtle shells is most extreme in sea turtles. Microscopy-based analyses indicate sea turtle shells are capable of supporting diverse epibiotic communities including numerous diatom species and epizoic macrofauna (e.g., barnacles, crustaceans) [12–15]. Although the nature of these communities appears to be influenced by both sex and species, the overall character of sea turtle epibiont assemblages remains relatively poorly understood, including to what degree shell microbial communities are impacted by geographic location [14,15]. In comparison to sea turtles, epibiotic communities of freshwater turtles are even less understood, although algal and microbial coverage can be similarly extensive. There is some evidence that turtle shell algal assemblages are variably reflective of local microbial communities. Observations of green algal growth on freshwater turtles date at least to the late 1800s [16], and the selective nature of *Basicladia* green algal growth on different species of turtles has been reported since the early 1900s [17,18]. Seasonal variation in algal colonization has also been reported, likely due to the timing of the shedding of the keratinous outer layer of shell scutes [19]. More recently, some support for broad geographic influence in *Luticola* diatom species composition was reported on the shells of common snapping turtles (*Chelydra serpentina*), suggesting a pattern similar to that found in the green algal epibiont *Basicladia* [20].

Nearly all previous investigations into turtle shell microbial communities have been microscopy-based. Simultaneously, microscopy techniques place de facto limitations on the accuracy and taxonomic level of microbial community disambiguation. These reports have focused on relatively large (e.g., filamentous green algae [18,19]) or more easily identifiable (e.g. diatoms [15,20]) eukaryotic taxa. Yet, even with seemingly morphologically discrete taxa such as diatoms, morphology-based analyses may result in underassessment of microbial diversity compared to molecular approaches [21,22]. Currently available molecular metabarcoding techniques provide fuller and more nuanced insight into prokaryotic and eukaryotic microbial community structure and ecology. As one example, recent efforts applying high-throughput sequencing (HTS) metabarcoding techniques to marine turtle shells uncovered cryptic diatom diversity, and supported that microscopy-based diversity estimates of turtle shell-associated microbial communities are likely to be low compared to molecular-based estimates [21]. Similarly, a single report thus far utilized HTS metabarcoding to characterize prokaryotic microbial communities of a freshwater turtle species, the Australian Krefft’s river turtle (*Emydura macquarii krefftii*) [23]. Among other findings, this report documented a prevalence of Cyanobacteria, Deinococcus-Thermus and Chloroflexi taxa on the shell and other external surfaces, as well as differing microbiome composition between shell areas with and without algal coverage. Together, these reports indicate that our understanding of turtle shell microbial communities may greatly benefit from the expanded application of molecular techniques.

In the present report, we characterize the prokaryotic and eukaryotic microbial communities present on the shells of the common and widespread freshwater turtle species *Trachemys scripta* (Thunberg in Schoepff, 1792) (i.e., the common red-eared slider) based on HTS 16S and 18S rDNA metabarcoding. Our data set includes turtles sampled from varied habitats primarily in central and western Oklahoma in the United States, but ranging to southeast Iowa. Our analyses primarily focus on 1) the taxonomic structure of shell-associated microbial communities, 2) microbial diversity comparisons among and between carapace (upper) and plastron (lower) scute surfaces, and 3) comparison between turtle shell and corresponding local environmental microbial communities, including for several bacterial taxa known to contain human pathogenic species. We also compare shell-associated microbial communities of *Trachemys scripta* samples to a shared-site, single collection of the ecologically and morphologically similar river cooter (*Pseudemys concinna* Le Conte 1830) to evaluate the potential for cross-species differences in shell-associated microbial communities. Our analyses add to a small but growing body of data characterizing turtle shells as a unique substrate for microbial colonization and ecology.

## Methods and materials

### Site selection and sample collections

**Fig 1:**
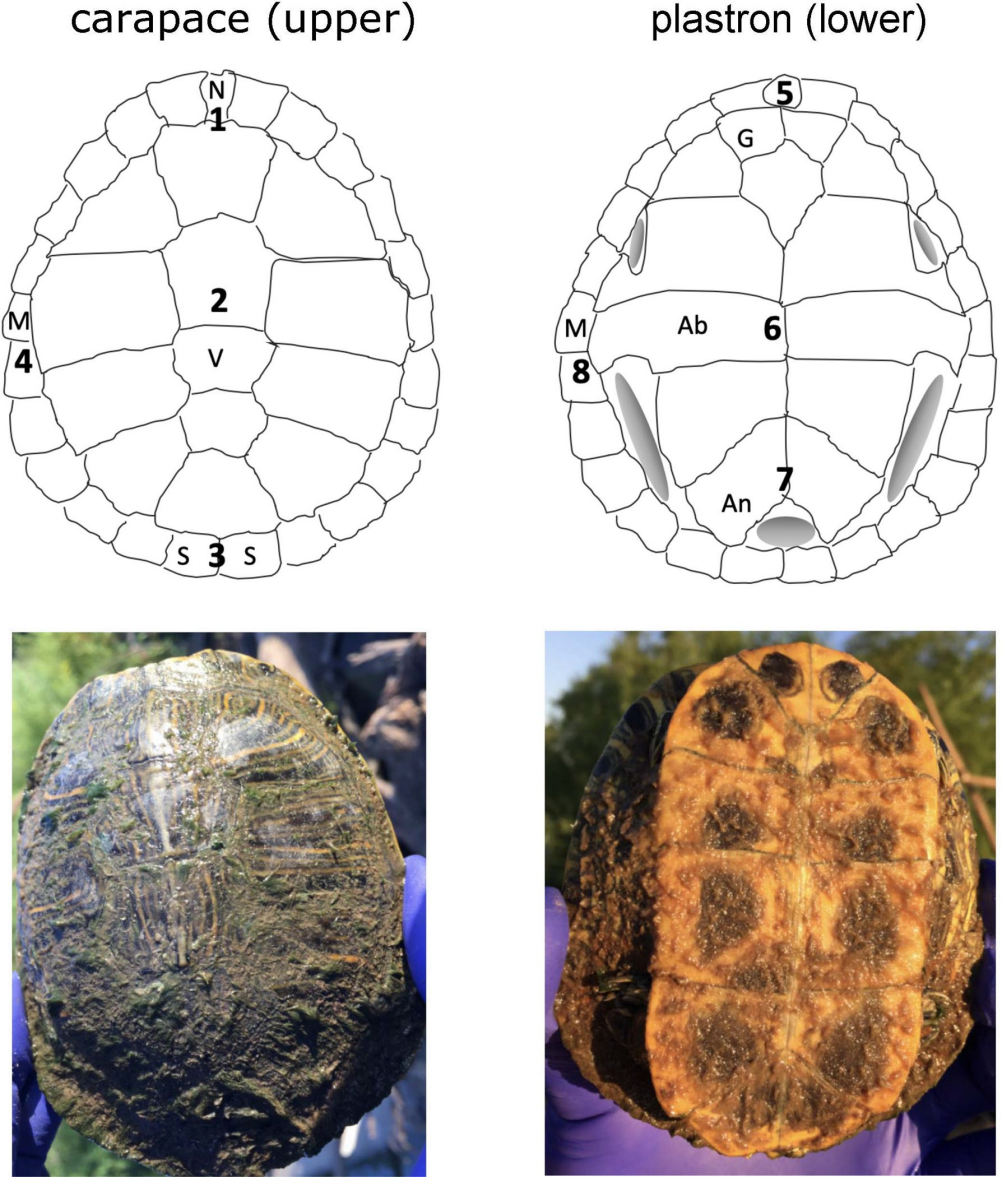
Scute sampling locations 1–8 for carapace (upper) and plastron (lower) shell surfaces, and pictures of representative sample of *T*. *scripta*. Scute abbreviations: Ab = abdominal; An = anal; G = gular; M = marginal; N = nuchal; S = supracaudal.

Eight sites were selected and sampled from June–September 2019, representing several habitats primarily in central Oklahoma, but ranging to SW Oklahoma and SE Iowa. Sites included large and small ponds, river sloughs and creeks, and a reservoir (**Table 1**). Two sites were sampled twice, at two different time points. At each site, resident turtles were live-trapped using wire-mesh/PVC basking traps (http://www.turtle-trap.com/) baited with canned sardines, with traps left overnight and for periods of no longer than 24 hours. Microbial community samples of ca. 50–125 mm^3^ were collected from 8 scutes per captured turtle (**Fig 1**) using a dental calculus scraper sterilized with 70% ethanol prior to collecting each sample. For the two sampled turtles at the “Altus large pond site” (ALP), four samples were collected from carapace scutes only, since detectable/extractable microbial growth was not present on plastron surfaces (this appeared to be due to recent shedding of plastron scute outer layers). While some collection methods employ a pure-water rinse of sampled surfaces prior to microbial collection [for example 24,25], we excluded this step so that sampled microbial communities reflected immediately present microbes and were not affected by loss of loosely associated resident cells or species. This may have resulted in sampling of some water-column associated microbes. However, we believe the impact of on our results this was likely negligible for two reasons: 1) sampled microbial communities were typically relatively dense, and so would be assumed to exclude most transient microbes, and 2) scrape samples taken from wet but ‘clean’ scutes (visibly lacking microbial growth), which would have included microbes from local water, did not contain measurable levels of DNA upon extraction. All trapped and sampled turtles were notched using unique pairs of lateral scutes, and were released unharmed on site after minimal handling. Microbial scrape samples were also collected from 2–3 shallowly submerged (ca. 5–15 cm) environmental substrates (primarily wood and rock/concrete) at each site. At each site, environmental and turtle scute scrape samples were collected on the same date, with the exception that environmental samples associated with the first turtle collection date of the “North Canadian River slough” site were collected ca. two weeks after turtle collection (10 July 2019 vs. 28 June 2019). Scrape samples were stored in ca. 0.5 ml 95% ethanol and maintained at -20° C for no longer than one month prior to DNA extraction. All turtle capture methods and field practices were approved prior to commencing field work by the University of Central Oklahoma Institutional Animal Care and Use Committee (UCO IACUC application #19005). Scrape samples were collected under a Scientific Collector’s Permit (ID #6233112) issued through the Oklahoma Department of Wildlife Conservation to Dr. Matthew Parks, with the exception of samples at the “Mississippi slough” site, which were collected with permission from Des Moines County Conservation and under the supervision of Mr. Jim Steer. Collections made on private properties were undertaken with verbal permission from property owners.

**Table 1:** Site locations, descriptions and sampling dates for turtle collections.

Site (abbreviation)	County, State	Latitude, longitude	Description	Sampling date(s) (all 2019)	# Turtles sampled for current study
N Canadian slough (NCS)	Oklahoma, Oklahoma	35.574, -97.275	Slow-moving side channel of N Canadian River	28-June, 08-September	2, 2
Chisholm Creek (CC)	Oklahoma, Oklahoma	35.653, -97.528	Pool/logjam in small wooded creek	02-July	2
Guthrie small pond (GSP)	Logan, Oklahoma	35.859, -97.319	Small (ca. 10^−3^ km^2^) wooded pond	03-July	2
Shell Creek tributary (SCT)	Canadian, Oklahoma	35.518, -97.786	Slow-moving tributary of N Canadian River	12-July	1
Yukon large pond (YLP)	Canadian, Oklahoma	35.524, -97.797	Private large pond (ca. 0.07 km^2^) in floodplain of N Canadian River	13,14-July, 15-September	2, 2
Mississippi slough (MS)	Des Moines, Iowa	40.724, -91.124	Sullivan Slough area of Mississippi River backwater	22-July	2
Overholser Reservoir (OR)	Oklahoma, Oklahoma	35.512, -97.667	Marsh area of large (ca. 6.5 km^2^) reservoir	12-August	2
Altus large pond (ALP)	Jackson, Oklahoma	34.635, -99.397	Private large farm pond (ca. 0.1 km^2^)	01-September	2

### DNA extraction and sample selection

Preserved scrape samples were centrifuged at 16,300×g for 3 minutes, and ethanol decanted prior to extraction. Pelleted material was resuspended in 410 μl lysis buffer PL1 (Takara Bio USA, Mountain View, California) and briefly vortexed to mix. Samples were then exposed to three sequential cellular disruption conditions: 1) freeze-thaw: 10 minutes incubation at -80° C, followed by 10 minutes incubation at 60° C, followed again by 15 minutes incubation at -80° C; 2) mechanical disruption: 60 seconds sonication with a Fisher Scientific FS20D Sonicator; 3) enzymatic digestion: 20 minutes incubation at 60° C with 20 μl proteinase K and 10 μl RNase A. DNA was subsequently extracted using the NucleoSpin Plant II DNA extraction kit and protocol (Takara Bio USA, Mountain View, California). Resulting DNA concentrations and A_260_/A_280_ ratios were measured using a Nanodrop spectrophotometer (Thermo Fisher Scientific). A total of 94 DNA extractions (71 scute + 23 environmental samples) were chosen for subsequent amplifications based on DNA quality and quantity, collection site and location of corresponding scute on the shell. All eight targeted scutes were selected from four *T*. *scripta* and one *P*. *concinna* turtles. Extractions from carapace location 3 and plastron location 8 were utilized from 16 additional *T*. *scripta* turtles. Two to three environmental DNA extractions from each site and collection date were also used in amplifications.

### PCR amplifications and sequencing

A two-step PCR amplification strategy was used to amplify 16S and 18S loci separately and in parallel and prepare samples for sequencing on the Illumina MiSeq platform. Primer design was based on the methods of [26], with the main exception that unique in-line indices were shifted to primers used in step 1 PCR. We found this slight alteration to be 1) more economical (all resulting primers were less than 60 base pairs in length), 2) simpler for downstream bioinformatic parsing (index combinations are always recovered in the same orientation on paired-end sequence reads), and 3) less prone to sample mix-up (since all samples are uniquely labeled in the first, rather than second, PCR amplification). Step 1 PCR primers targeted the V3-V4 16S and V8-V9 18S regions, using the Pro341F/Pro805R [27] and V8f/1510r [28,29] primer sets, respectively. Step 1 primer tails consisted of variable in-line indices of 2–7 base pairs in length and sequence complementary to Illumina sequencing primers. Step 1 amplifications were composed of 1.5 μl millipore-filtered water, 1.0 μl BSA (New England Biolabs), 2× Taq MasterMix (New England Biolabs), 0.5 μl each forward and reverse 10 μM primers, and 4 μl full or diluted DNA extraction (representing ca. 10–100 ng DNA). PCR conditions for step 1 16S amplifications were: 95° C for 2 minutes, 25 cycles of 95° C for 15 seconds/65-55° C for 30 seconds (1° C decrease per cycle for first 10 cycles)/68° C for 30 seconds, followed by a final extension at 68° C for 5 minutes. PCR conditions for step 1 18S amplifications were: 95° C for 2 minutes, 25 cycles of 95° C for 15 seconds/58° C for 30 seconds/68° C for 30 seconds, followed by a final extension at 68° C for 5 minutes. All step 1 PCR amplifications were performed in triplicate in 96-well plate format, with a negative control sample (with millipore-filtered water replacing input DNA extraction) placed in a different internal well in each plate. Full details of the PCR indexing scheme and all primer sequences used are available in **S1 Appendix**.

For each sample, 5 μl of product from each of the three replicate step 1 amplifications was pooled and used as an input source for step 2 PCR amplifications. Step 2 PCR amplifications utilized primers complementary to step 1 primer tails and containing sequence complementary to Illumina flowcell-bound oligonucleotides, and were performed separately for 16S and 18S amplification products. Step 2 amplifications for each sample contained 7.5 μl 2× PCR MasterMix, 1 μl each of 10 μl 10 μM forward and reverse primers, 4.5 μl millipore-filtered water, and 1 μl product from pooled step 1 PCR amplifications. PCR conditions for step 2 amplifications were: 95° C for 2 minutes, 10 cycles of 95° C for 15 seconds/55° C for 30 seconds/72° C for 30 seconds, followed by a final extension at 72° C for 5 minutes.

PCR products were checked for size and intensity using a 1% agarose gel stained with GelRed (www.biotium.com). All step 2 PCR reactions (94×16S + 94×18S = 188 total) were pooled ca. equimolarly and purified using Agencourt Ampure XP beads (www.beckman.com), at a ratio of 0.8:1 product:beads to remove residual primers and potential primer-dimers. Sequencing was completed at the University of Central Oklahoma on an Illumina MiSeq platform, using standard procedures with a MiSeq Reagent Kit V3 (600 cycle) (www.illumina.com) to produce 300 base pair paired-end reads.

### Data analyses

Samples were first parsed from raw sequencing data based on 5´ and 3´ in-line index sequences using custom Unix scripting, and index and primer sequences were removed from forward and reverse reads with Cutadapt ver. 2.10 [30]. Subsequent visualization, denoising, and diversity and taxonomic analyses were completed using QIIME 2 ver. 2019.10 [31] separately and in parallel for both 16S and 18S sequence pools, and largely followed the “moving pictures” tutorial guidelines (https://docs.qiime2.org/2019.10/tutorials/moving-pictures/). Sequences were denoised with DADA2 [32], using forward and reverse reads trimmed to 277 and 186 bases for 16S, and 273 and 218 bases for 18S sequences based on quality score profiles (cutoff determined as the 5´-most position before the 25^th^ percentile first dropped below Q = 30). Resulting denoised reads were *de novo* clustered at 97% identity cutoff and filtered for a minimum frequency of 100 and presence in at least two samples, to decrease potential artifacts of sequencing error. Taxonomic classification training was performed using the pre-trained Naïve Bayes classifier (classify-sklearn) and the feature-classifier QIIME2 plugin, and using SILVA [33] v132 99% 16S and 18S databases with targeted amplified regions extracted using the “microbiome_helper” guidelines (https://github.com/LangilleLab/microbiome_helper/wiki/Creating-QIIME-2-Taxonomic-Classifiers). Taxonomic accuracy in the resulting databases was validated using the “taxa_sanity_check” pipeline (https://github.com/gavinmdouglas/taxa_sanity_check). All features affiliated to “chloroplast”, “mitochondria”, or residing within the 18S “Metazoa: Tetrapoda” lineage were filtered from the dataset. A phylogenetic tree was generated for 16S and 18S sequences using the align-to-tree-mafft-fasttree function of QIIME2. Sampling depth (i.e., frequency of features per sample) for subsequent diversity core-metrics was determined as the highest sampling depth that allowed inclusion of 100% (i.e., 94/94) of samples. This value was 2,555 for 16S samples and 2,586 for 18S samples. Alpha rarefaction curves were used to validate inclusion of ≥90% of 16S and 18S microbial diversity at minimal sampling depths based on three applied alpha diversity metrics: 1) Shannon diversity, 2) Faith’s Phylogenetic diversity, and 3) Observed OTUs (16S and 18S alpha rarefaction curves and tables for these metrics are available in **S2 Appendix**).

### Diversity and abundance analyses

Alpha and beta diversity analyses were completed for turtle shell and environmental communities to assess variability in microbial communities across three primary comparisons: 1) plastron vs. carapace, 2) plastron or carapace vs. environment, and 3) individual plastron and carapace scute locations. For plastron vs. carapace comparisons, we used all samples from *T*. *scripta* scute 3 (carapace) and scute 8 (plastron) samples. For plastron and carapace vs. environmental samples, we used either all scute 3 or all scute 8 samples from sampled *T*. *scripta*, and all wood and rock/concrete environmental samples. Scute locations 3 and 8 were chosen for these analyses because they provided the most complete representation of carapace and plastron surfaces based on DNA extraction and PCR success. For individual plastron and carapace scute comparisons, we used scute locations 1–8 on the four *T*. *scripta* collections from which all of these scute locations were sampled. Alpha diversity was assessed using three metrics: Shannon diversity, Faith’s phylogenetic diversity and Observed OTUs. Significance for alpha diversity comparisons was assessed using linear mixed effect modeling in the lme4 package for R [34] and the Satterthwaite approximation to account for small sample size [35]. Beta diversity was estimated using four dissimilarity indices: Jaccard, Bray-Curtis, and unweighted and weighted UniFrac. Significance in beta diversity comparisons for Jaccard and Bray-Curtis metrics was assessed through PERMANOVA tests using adonis2 in the R vegan package [36]; p-values were corrected using a Benjamini-Hochberg correction [37]. In mixed effect models, study site was applied as a fixed effect in all comparisons; turtle ID was applied in all comparisons as a random effect (alpha diversity) or strata (beta diversity), as our sampling involved multiple scutes per turtle captured and multiple turtles per site. For both alpha and beta diversity analyses, we first checked for significant differences in microbial communities between soft (wood) and hard (rock/concrete) environmental substrates. We recovered significant differences between these sample types in Faith’s Phylogenetic diversity, Observed OTUs, Jaccard, Bray-Curtis and unweighted UniFrac measures for 18S communities only. For these metrics in 18S alpha and beta diversity analyses we subsequently included environmental substrate type (wood vs. rock/concrete) as a fixed effect in statistical analyses. For all other analyses, wood and concrete samples were classified together as environmental samples. We checked for normality and the presence of outliers by visual inspection of residual plots (alpha diversity metrics) and NMDS plots (beta diversity ordination) for all applied metrics and data subsets. Alpha and beta diversity plots were created using the ggplot2 [38] and phyloseq [39] and R packages.

ANCOM differential abundance analyses [40] were completed for 16S and 18S taxonomic diversity by comparing environmental (wood and rock/concrete) and scute samples (all *T*. *scripta* scute 3 and 8 samples) across sampling locations. These analyses were completed for Class, Order, Phylum and Genus, (i.e., taxonomic levels 3–6), since ANCOM analysis may be impacted at low taxonomic levels by high proportions of differentially abundant taxa [40]. Last, we used the same group of samples to evaluate the distribution of ten bacterial genera and families, which include human-pathogenic species, recovered in our 16S sequencing data: *Aeromonas* (Aeromonadaceae), *Clostridium* (Clostridiaceae), *Enterococcus* (Enterococcaceae), *Francisella* (Francisellaceae), *Legionella* (Legionellaceae), *Leptospira* (Leptospiraceae), *Neochlamydia* (Parachlamydiaceae), *Plesiomonas* (Enterobacteriaceae), *Pseudomonas* (Pseudomonadaceae), and *Vibrio* (Vibrionaceae).

### Data availability

16S and 18S sequence data, with indices and primers removed and parsed by sample, is available through NCBI SRA (BioProject PRJNA639844).

## Results

### DNA extractions, sequencing and denoising

DNA concentrations from selected extractions ranged from 1.9–360.7 ng/μl (average ± standard deviation = 82.6 ± 89.6 ng/μl). We recovered a total of 10,463,228 raw sequence reads, with 48,779 ± 17,671 (range = 11,460–103,674) average raw read pair counts for 16S sequences and 52,078 ± 15,049 (range = 9,121–81,123) for 18S sequences. No correlation was found between 16S and 18S per-sample raw read counts (y = 0.0494x + 49667, R^2^ = 0.0034); however, across all samples, average read counts for 16S and 18S environmental samples were slightly higher (ca. 10–18%) than for turtle scute samples.

**Fig 2:**
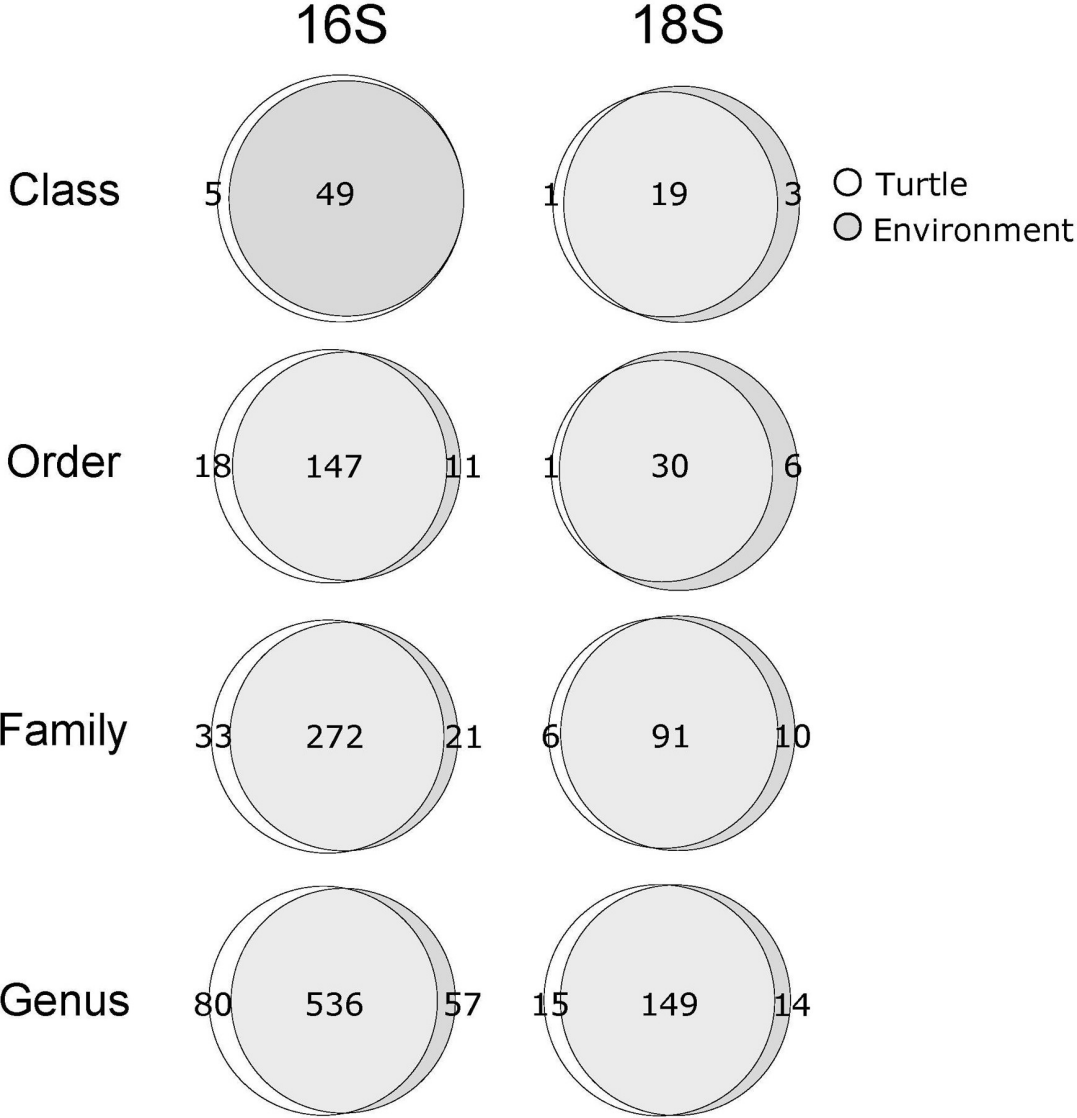
Venn diagrams of recovered taxon counts across all 16S and 18S turtle and environmental samples at the Class, Order, Family and Genus level. Counts of shared and unique taxonomic assignments recovered in environmental and turtle samples are indicated for 16S (left column) and 18S (right column) communities. Data from *T*. *scripta* and *P*. *concinna* is included, as inclusion of *P*. *concinna* samples had negligible impact on counts of recovered taxonomic assignments.

After quality trimming, denoising and clustering, we recovered 1,734 and 315 total 16S and 18S OTUs across all samples; the majority of assignments of OTUs to identified sequence variants (ca. 61.5%) had relatively high confidence (confidence ≥ 0.95). At the class and genus level, turtle and environmental samples contained from 49–616 and 20–154 assigned taxa for 16S and 18S samples, respectively (**Fig 2**). On average, ca. 18% of these assignments were unique to either turtle or environmental samples.

### Microbial community taxonomic structure

**Fig 3:**
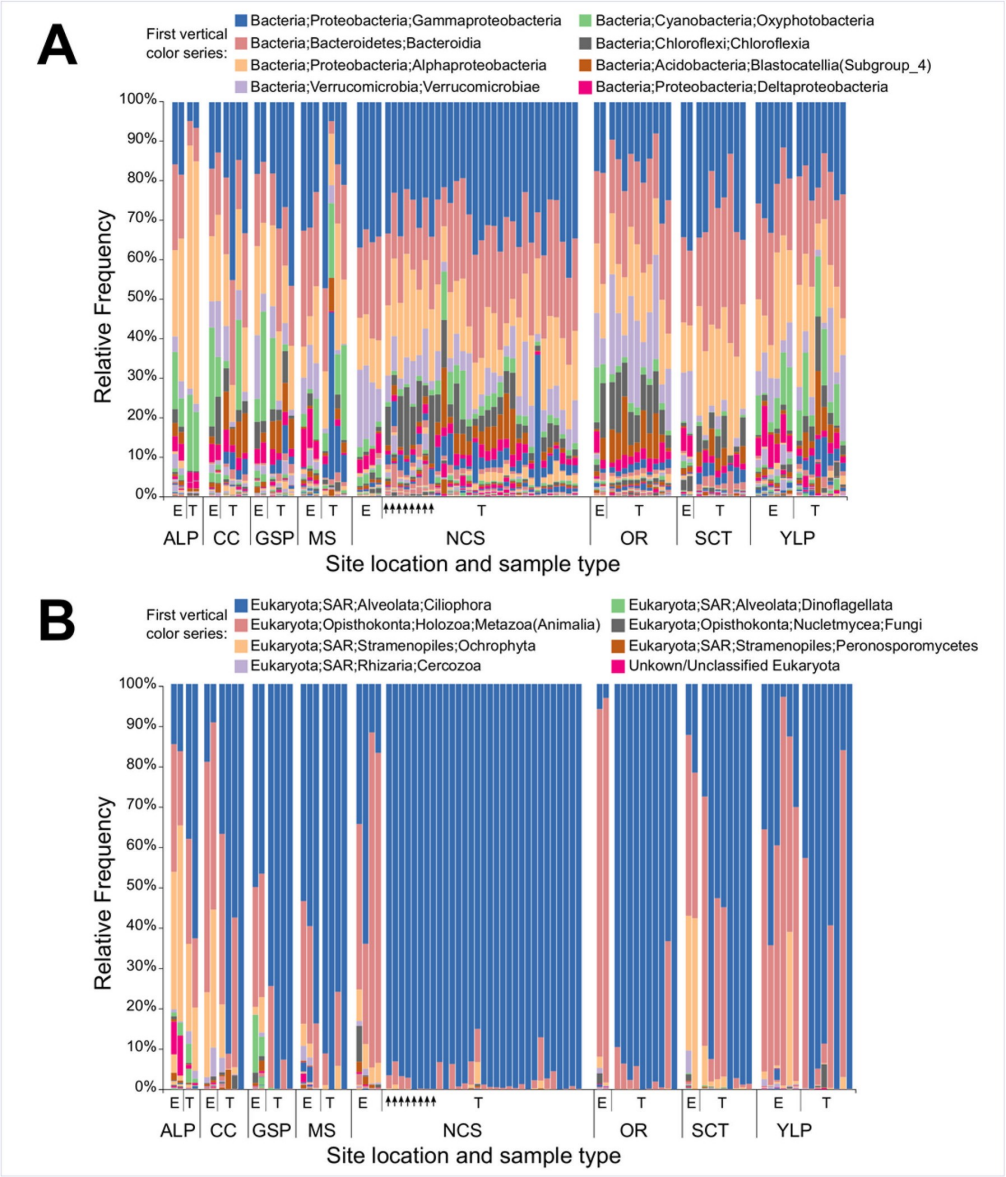
Taxonomic diversity of turtle scute and environmental microbial communities. (A) Taxonomic affiliation of 16S communities at the Class level; (B) Taxonomic affiliation of 18S communities at the Order level. Samples are grouped by site, and separated within site by environmental (E) and turtle (T) samples. Vertical arrows indicate scute microbial communities sampled from *P*. *concinna* at the NCS site.

While recovered microbial communities were taxonomically diverse, both 16S and 18S communities were dominated by several taxa in a majority of samples. For example, 64/71 scute and 22/23 environmental 16S communities were composed of over 50% Alpha- and Gamma-Proteobacteria, and Bacteroidia (Bacteroidetes) (**Fig 3**). Several other bacterial taxa, including Verrucomicrobiae (Verrucomicrobia), Oxyphotobacteria (Cyanobacteria), and Chloroflexia (Chloroflexi) were also strongly represented in many of the 16S scute and environmental communities, while many scute samples also had substantial representation of Blastocatellia (Acidobacteria). Similarly, a strong majority (66/71) of 18S scute communities were composed of over 50% ciliophoran alveolates (SAR) (**Fig 3**). Identified alveolates were predominantly Peritrichia ciliates, although carapace scute samples from the single *T*. *scripta* turtle collected at the “Shell Creek tributary” site were compositionally dominated (>50%) by Haptoria ciliates and Chromadorea metazoans. 18S environmental samples were generally dominated by metazoan Opisthokonta and ciliophoran alveolates, with substantial contributions from Ochrophyta stramenopiles in many samples. Neoptera, Oligochaete and Podocopa opisthokont taxa, and Peritrichea and Heterotrichea alveolates, were frequently strongly represented in environmental samples. Complete taxonomic frequencies for all 16S and 18S communities at the Class, Order, Family and Genus level are available in **S3 Appendix**.

### Alpha and beta diversity comparisons

**Fig 4:**
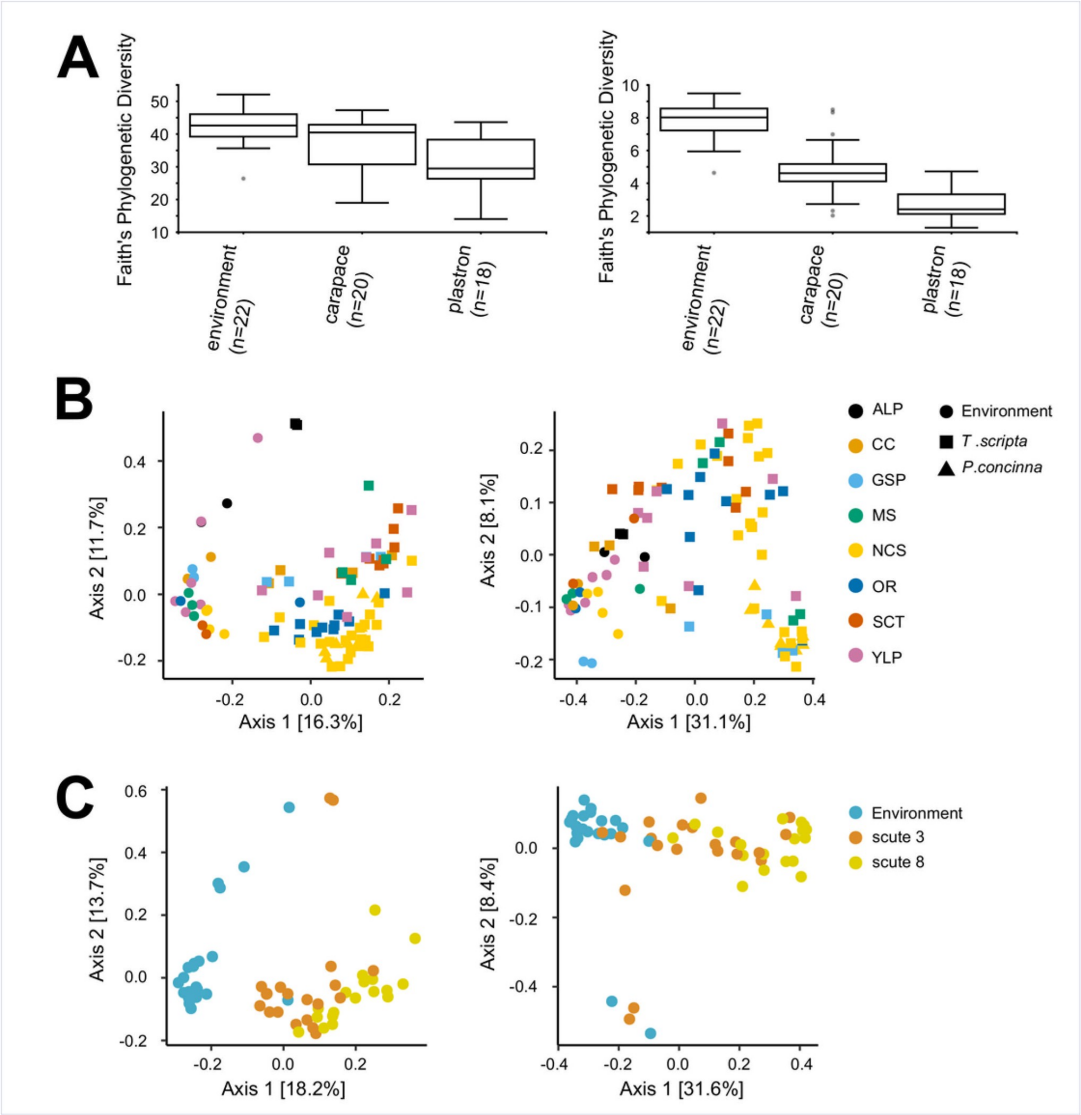
Representative alpha and beta diversity comparisons between environmental, carapace and plastron microbial communities. A) Alpha diversity (Faith’s Phylogenetic diversity) estimates for 16S (left) and 18S (right) environmental, *T*. *scripta* carapace (scute 3) and *T*. *scripta* plastron (scute 8) communities. B) Beta diversity (unweighted UniFrac) PCoA plots for all wood and rock/concrete environmental samples, and all *T*. *scripta* and *P*. *concinna* scute samples. Sample provenance is indicated by color and shape (see legend). C) Beta diversity (unweighted UniFrac) PCoA plots for wood and rock/concrete environmental samples, and *T*. *scripta* carapace (scute 3) and plastron (scute 8) samples. Sample provenance is indicated by color (see legend). Corresponding plots for all applied alpha and beta diversity metrics are available in **S4 Appendix**.

In alpha diversity comparisons, we recovered significant differences across nearly all applied metrics for *T*. *scripta* carapace vs. plastron, carapace vs. environment, and plastron vs. environment communities (Table 2). We did not recover significant differences in comparisons of individual scute locations on either the carapace or plastron of *T*. *scripta* shells (**Table 2**). For both 16S and 18S samples, carapace communities had a higher diversity than plastron communities, while both were lower than environmental samples (**Fig 4 and S4 Appendix**). Study site was a significant effect for some alpha diversity metrics across all comparisons except plastron vs. environment and carapace scute location (i.e., scutes 1–4), although it was never recovered as a significant effect across all metrics for any comparison (**Table 2**).

**Table 2:** Results of significance testing for alpha and beta diversity metrics in *T*. *scripta* scute and environmental sample comparisons. Alpha and beta diversity test corrected p-values are shown in scientific notation; significant results (corrected p-value ≤ 0.05) are indicated in **bold**. p-values shown are corrected using either a Satterthwaite approximation (alpha diversity) or Benjamini-Hochberg correction (beta diversity). “Substrate” was included as a fixed effect for linear mixed effect modeling of 18S Shannon diversity, Jaccard, Bray-Curtis and unweighted UniFrac, since we recovered significant differences between wood- and rock/concrete-associated microbial communities for these metrics (see Methods and Materials). env = shallow water environmental samples.

Comparison	Fixed effects	Alpha diversity			Beta diversity			
		Shannon	Faith	Obs. OTUs	Bray-Curtis	Jaccard	Unw-UniFrac	W-UniFrac
**16S**								
carapace vs. plastron	carapace vs. plastron, study site	**8.0E-5**	**2.7E-4**	**1.7E-5**	**1E-3**	**1E-3**	**1E-3**	**1E-3**
		**9.0E-5**	**1.6E-5**	1.9E+0	**1E-3**	**1E-3**	**1E-3**	**1E-3**
carapace vs. environment	env vs. turtle, study site	**3.7E-4**	**1.2E-2**	**1.3E-3**	**1E-3**	**1E-3**	**1E-3**	**1E-3**
		7.4E-2	9.0E-2	1.3E-1	**1E-3**	**1E-3**	**1E-3**	**1E-3**
plastron vs. environment	env vs. turtle, study site	**2.5E-6**	**1.3E-4**	**4.4E-6**	**1E-3**	**1E-3**	**1E-3**	**1E-3**
		3E-1	3.4E-1	4.8E-1	**1E-3**	**1E-3**	**2E-3**	7.7E-2
scutes 1–4, all vs. all	scute location, study site	4.9E-1	3.1E-1	4.3E-1	5.3E-1	6.2E-1	6.8E-1	1.6E-1
		8.1E-1	3.5E-1	6.6E-1	**1E-3**	**1E-3**	**1E-3**	**1E-3**
scutes 5–8, all vs. all	scute location, study site	4.7E-1	4.3E-1	7.8E-1	7.1E-1	7.8E-1	7.4E-1	2.8E-1
		1.5E-1	**2.0E-3**	**3.0E-2**	**1E-3**	**1E-3**	**1E-3**	**1E-3**
**18S**								
carapace vs. plastron	carapace vs. plastron, study site	8.3E-2	**6.3E-6**	**5.5E-6**	**1E-3**	**1E-3**	**1E-3**	**1E-3**
		**5.7E-5**	5.5E-2	1.0E-1	**1E-3**	**1E-3**	**1E-3**	**3E-3**
carapace vs. environment	env vs. turtle, study site, substrate	**2.3E-3**	**1.1E-6**	**2.1E-8**	**1E-3**	**1E-3**	**1E-3**	**1E-3**
		**1.1E-2**	6.7E-2	**2.9E-2**	**1E-3**	**1E-3**	**1E-3**	**1E-3**
		5.6E-2	NA	NA	5.4E-1	5.9E-1	4.1E-1	NA
plastron vs. environment	env vs. turtle, study site, substrate	**3.8E-11**	**2.1E-10**	**3.7E-13**	**1E-3**	**1E-3**	**1E-3**	**1E-3**
		1.8E-1	9.5E-1	6.7E-1	**1E-3**	**1E-3**	**1E-3**	**1E-3**
		**3.9E-2**	NA	NA	2.1E-1	2.0–1	4.0E-1	**NA**
scutes 1–4, all vs. all	scute location, study site	2.3E-1	8.1E-1	1.4E-1	2.8E-1	3.9E-1	4.0E-1	5.7E-1
		5.1E-1	5.4E-1	5.3E-1	**1E-3**	**1E-3**	**1E-3**	**5E-3**
scutes 5–8, all vs. all	scute location, study site	5.2E-1	8.3E-2	1.3E-1	7.2E-1	6.9E-1	5.8E-1	5.9E-1
		7.9E-1	7.9E-1	6.8E-1	**2.7E-2**	**2.1E-2**	**2E-3**	**2.1E-2**

Beta diversity was noticeably differentiated between environmental and turtle shell communities, including for *P*. *concinna* scute samples, which largely clustered with same-site *T*. *scripta* samples (**Fig 4 and S4 Appendix**). Comparisons were reflective of alpha diversity results, supporting differentiation between carapace, plastron and environmental communities. Significant differences in beta diversity were recovered across all metrics for both 16S and 18S comparisons of carapace vs. plastron, carapace vs. environment and plastron vs. environment (**Fig 4 and Table 2**); no significant differences were recovered across individual plastron or carapace scute locations (**Table 2**). In beta diversity analyses, “study site” (i.e., location of sample collection) was a significant factor across all metrics and comparisons, with the exception of the weighted UniFrac metric for 16S plastron vs. environment comparison (Table 2).

### Differential abundance of microbial taxa

We recovered 49 taxa with significant differential abundance between turtle (*T*. *scripta* scutes 3 and 8 combined) and environmental (wood and rock/concrete combined) samples for 16S and 18S microbial communities, across the different taxonomic levels (Class, Order, Family and Genus, respectively) (**Table 3**). Eleven 16S genera were more abundant on turtle shells. These results were generally consistent across sampling sites, except for seven genera which were either less abundant on turtle shells or not recovered in sequencing data from the ALP site, and one genus which was less abundant on turtle shells compared to environmental samples at each of the OR and MS sites. The *Deinococcus* bacterial lineage (belonging to the Deinococcus-Thermus phylum) was the only 16S lineage recovered with differential abundance across all taxonomic levels examined, and was in all cases more abundant in turtle scute samples than in environmental samples. Three differentially abundant bacterial genera (*Synechococcus* PCC-7902, *Rubrivirga*, *Paracoccus*) were recovered exclusively on turtle shells. Four 18S genera (*Epistylis*, *Tokophrya*, *Opercularia*, *Heliophrya*) were more abundant on turtle shells compared to environmental samples; none of these genera were exclusive to turtle shells. No significant differences were observed in the abundance of any of the ten potentially pathogenic genera/families identified. All of these genera were nonetheless recovered from turtle shell microbial communities, and two genera (*Enterococcus*, *Francisella*) were recovered exclusively on turtle shells. Full taxonomic details of 16S and 18S ANCOM analyses are available in **S5 Appendix**.

**Table 3:** Number of taxa with significantly different abundance in turtle shell- versus environmental sample-associated 16S and 18S communities. Counts are shown for taxonomic levels Class through Genus. Numbers in parentheses indicate numbers of instances of significantly higher abundance taxonomic assignments in (turtle samples, environmental samples) across sites.

Microbial community	Taxonomic level			
	Class	Order	Family	Genus
16S	1 (1, 0)	12 (8, 4)	8 (7, 1)	14 (11, 3)
18S	1 (1, 0)	3 (1, 2)	2 (2, 0)	8 (4, 4)

## Discussion

Both freshwater turtle species and the aquatic environments they inhabit are increasingly imperiled by anthropogenic drivers. Nearly two-thirds of known turtle and tortoise species are currently classified from vulnerable to fully extinct [41], with harvest and urban development among the top threats. This level of threat is currently greater than that faced by birds, mammals, fish or amphibians [42,43]. Similarly, human activity is impacting freshwater ecosystems worldwide both directly and indirectly through pollution, overharvest, watershed alteration and climate change [44]. Since microbial communities are clearly linked to and indicative of both organismal health [45] and environmental condition [46,47] in freshwater habitats, it is increasingly critical to understand associated ecosystem-wide microbial communities and processes as part of management and conservation plans. Our data contribute to the understanding of shell-associated microbial communities in a common and widespread freshwater turtle species, the red-eared slider (*T*. *scripta*), in the context of microbial community taxonomic characterization, uniqueness, and potential as a bio-indicator of overall environmental status. The application of a metabarcoding strategy provides finer-scale resolution of shell-associated microbial constituencies compared to microscopy-based efforts, and our study is the first to describe full 18S microbial communities on external turtle shell surfaces. Inclusion of a single sampled individual of the river cooter (*P*. *concinna*) also provides insight into cross-species comparisons between two morphologically and ecologically overlapping turtle species [48,49].

In our sampling, we recovered highly diverse communities from both turtle shell substrates and shallow-water environmental substrates across a total of eight collection sites, representing different habitats and separated by up to ca. 1000 km. Our samples contained over 1,200 high confidence taxonomic affiliations within over 2,000 recovered 16S and 18S taxa in total, representing hundreds of microbial taxa. Conversely, turtle scute samples were also often dominated by one to several taxa, including the Bacteroidia and Alpha- and Gamma-proteobacteria bacterial classes, and Peritrichia ciliates. Although alpha-rarefaction curves supported that our analyses captured ≥90% of microbial OTU diversity at our sequencing levels, deeper sequencing would likely further facilitate understanding of microbial community structure in relation to the relatively numerous, uncommon 16S and 18S taxa recovered in our data.

Alpha diversity was consistently lower for both 16S and 18S communities across our sampled sites in *T*. *scripta* scute samples compared to shallow-water environmental samples. It is likely that reduced alpha diversity on turtle shells is influenced by regular drying periods during basking behavior, as red-eared slider (and river cooter) turtles commonly bask either partially or completely out of the water for extended periods of time [50]. Emersion activity has similarly been implicated as an anti-microbial-fouling mechanism in marine crab species [51]. In addition, shedding of the outer keratinous layer of scutes likely occurs on the order of one to several times per year, predominantly in warmer summer months [52], which should have the effect of largely ‘resetting’ microbial communities. We observed both basking behaviors and variable levels of scute shedding at sample sites and in captured turtles during sample collection. Nonetheless, ca. 8–12% of 16S and 18S taxa recovered from turtle shell communities were unique to turtle scutes. It is not clear whether these taxa are fully unique to turtle shells, since our environmental sampling was limited to only shallow-water submerged substrates. Assessment of these taxa in subsequent freshwater aquatic microbial sampling efforts may clarify these results. For microbial taxa recovered in our study as environment-specific, it is similarly unclear whether these taxa are potentially restricted from colonizing turtle shells by chemical or structural properties of turtle shell scutes, the presence or absence of associated microbial taxa, or abiotic conditions. It is possible that at least a portion of these taxa could persist on turtle shells under different conditions, for example lower frequency of basking-drying and/or shedding cycles, or could yet be recovered from turtle shells with wider within- or across-species sampling or deeper sequencing efforts.

For both alpha and beta diversity measures, we recovered significant differences in nearly all applied metrics when comparing carapace and plastron surfaces, but did not recover differences across individual carapace or plastron scute locations with any applied metrics. This suggests that carapace and plastron surfaces provide, at some level, fundamentally differing microbial environments. It is not immediately clear what factors most strongly influence carapace-plastron differences, however alternating exposure of carapace surfaces to sunlight and shade, and wet/dry cycling, expected during normal daily turtle movements, as well as mechanical disruption of plastron microbial communities (through scraping of plastron surfaces on environmental substrates) may interact in some way to favor elevated microbial diversity of carapace surfaces. Study site was more consistently recovered as a significant factor in beta diversity compared to alpha diversity. These results support that while overall levels of microbial diversity are expected to remain relatively consistent across the shells of *T*. *scripta*, at least within our sampling region, microbial species composition does appear to be influenced by site or habitat. Our results are reflective of [53], which support that alpha diversity of external microbiota may be determined by physical characteristics of aquatic host species, while beta diversity is more strongly influenced by local environmental factors. In-depth physical characterization of sampled habitats would enable understanding of the role of environmental variables (levels of dissolved organic matter, water temperature, pH, etc.) in microbial species composition of turtle shell surfaces in future studies. Our results further indicate that the dominant Phyla and Classes within microbial communities were generally stable across our sampling sites (**Fig 3**). In this regard, we also note that the most prominent 16S taxa recovered in our turtle shell samples are in relatively close alignment with those recovered from shells of the Australian Krefft’s river turtle [23] across taxonomic levels Class, Order and Family. We therefore suggest that microbial constituency for the shells of freshwater basking turtles is relatively stable across habitats at higher taxonomic levels, with diversity most likely concentrated at lower taxonomic levels (Genus, Species).

The lineages we recovered as significantly different in abundance in *T*. *scripta* shell vs. environmental samples may also in part reflect differing physical conditions. For example, the Deinococcus-Thermus phylum, recovered at all sites as more abundant on turtle shells, is considered an extremophilic group, with member taxa variably resistant to extreme temperatures, radiation, and desiccation [54]. These characteristics may support the ability to withstand high sun exposure and drying conditions associated with basking turtles. It is not immediately clear why these bacteria were not consistently recovered on the shallow-water substrates we sampled, and members of the Deinococcus-Thermus phylum previously have been reported in algae-associated microbial communities [55] among other habitats. Deinococcus-Thermus were also reported as common in external microbiomes of the Australian Krefft’s river turtle [23]. Further exploration specifically of the Deinococcus-Thermus phylum on freshwater turtle shells could yield insight into its diversity, and potentially patterns of co-speciation across the shells of different turtle species. Three bacterial genera recovered only on turtle shells in our data set (*Synechococcus*, *Rubrivirga*, *Paracoccus*) have all previously been recovered from environmental sampling, so it is probable that these taxa would be recovered at our sites with more extensive environmental sampling. Nonetheless, *Synechococcus* PCC-7902 was originally reported from polar bear [56] hair, which is similarly a keratin-based external surface of a semi-aquatic vertebrate animal. The 18S *Epistylis*, *Tokophrya*, *Opercularia* and *Heliophrya* genera are known as aquatic epibionts, and in some cases *Epistylis* in particular may have negative impacts on host health [57,58]. Most likely, these taxa were recovered as most abundant on turtle shells through a combination of factors, including relatively high silt and organic matter conditions common to our sampling sites [59,60], aggressive colonization of aquatic substrates [61], and potentially desiccation resistance [62]. *Tokophrya* may also form long-term, specific epibiont relationships [63], suggesting that this lineage, similar to the Deinococcus-Thermus phylum, may provide a unique opportunity to study epibiont adaptation and co-evolution in semi-aquatic system. Overall, the differential distribution of 16S and 18S taxa recovered in our data support that the turtle shell provides a unique niche for evolution in at least several microbial lineages, a trait that is likely shared with the external surfaces of other aquatic vertebrate taxa [64]. Dalla Valle et al. [65] demonstrated that the specific keratins composing the external shell surface of turtles also have relatively strong homology to those found in the crocodilian epidermis. Since the native and introduced geographic ranges of *T*. *scripta* overlap with several crocodilian species, including both the American alligator (*Alligator mississippiensis*) and crocodile (*Crocodylus acutus*), sampling of turtle and crocodilian external microbial communities in shared habitats could provide further insight into the specific role of turtle ecology and shell chemistry in determining resident microbial community assemblage.

Across the present study sites tested we recovered ten genera of bacteria that are known to contain human pathogens, although we did not test these samples specifically for pathogenicity. Two genera/families (*Enterococcus* (Enterococcaceae), *Francisella* (Francisellaceae)) were recovered solely from turtle shells, but these results were not significant in our analyses. In each of the remaining genera/families, abundance was variably higher in turtle shell and environmental samples. This suggests that while turtle shells could carry environmentally-present pathogenic bacteria, it is unlikely that they generally act to concentrate these taxa to high abundance in shell-associated microbial communities. It is also unlikely that turtle shell microbial communities could serve as effective early indicators for outbreaks of virulent bacterial strains in aquatic environments. Nonetheless, our data do support that freshwater turtle shells, similar to their marine counterparts [21], can serve as a biofilm ‘reservoir’ for a collection of microbial taxa, potentially including for pathogenic bacteria. As such, turtles may act as regional vectors during habitat selection and nest-/mate-searching movements [66].

Consideration of the shell-associated microbiota from a single *P*. *concinna* individual suggest that microbial communities may largely overlap between ecologically and morphologically similar turtle species (**Fig 4**). *P*. *concinna* and *T*. *scripta* microbial communities were similarly distant from environmental samples in both 16S and 18S alpha diversity (data not shown), and *P*. *concinna* scute samples were within the range of variation of *T*. *scripta* samples in beta diversity ordination (**Fig 4**). While a larger dataset would permit stronger comparisons between *T*. *scripta* and *P*. *concinna*, this likely would take considerably increased field sampling effort due to the disparity in abundance of these two species in Oklahoma [49] and likely throughout their ranges.

Finally, although our study focused primarily on *T*. *scripta* populations within Oklahoma, similar metabarcoding methods could easily be extended to other *T*. *scripta* populations and to other freshwater turtle species [23]. Red-eared sliders are both widely distributed in their native range in the eastern half of North America, and across every other continent except Antarctica through widespread introductions. Although considered highly invasive [67], as a widespread introduced species *T*. *scripta* also provides unique opportunities to assess microbial community variation and impact on both organismal and ecosystem health and functioning. While our results do not support that the shells of *T*. *scripta* act as complete bio-accumulators for aquatic environments, it is clear that they support diverse microbial communities that are not simple reflections of environmental microbial populations, and should prove a worthwhile area of focus for future studies in both microbial and vertebrate ecology.

## Supporting information

S1 Appendix Sample indexing scheme and primer sequences. This file contains the indexing scheme applied in PCR1 amplifications and sequencing for turtle scute and environmental sample, and sequences of PCR primers used in 16S and 18S PCR1 and PCR2 amplifications.
(PDF)

S2 Appendix Alpha rarefaction curves and associated tables for 16S and 18S sampling. This folder contains comma-delimited alpha rarefaction tables for 16 and 18S Faith’s Phylogenetic Diversity, Observed OTUs and Shannon diversity for all samples, as well as the corresponding alpha rarefaction curve images for these diversity indices for all samples. Identifying sample information is found in the final three columns of each rarefaction table.
(ZIP)

S3 Appendix Sample-specific feature counts for 16S and 18S taxonomic assignments. This folder contains comma-delimited tables of 16S and 18S sample feature counts corresponding to taxonomic classifications for taxonomic levels Class, Order, Family and Genus. Identifying sample information is found in the final three columns of each table.
(ZIP)

S4 Appendix Alpha and beta diversity plots. This file contains: 4.1) alpha diversity box plots for all alpha diversity metrics applied, for 16S and 18S environmental (wood and rock/concrete), *T*. *scripta* carapace (scute 3) and *T*. *scripta* plastron (scute 8) comparisons; 4.2) Beta diversity emperor plots for all applied beta diversity metrics, for all 16S and 18S environmental and scute samples; 4.3) Beta diversity emperor plots for all applied beta diversity metrics, for 16S and 18S environmental (wood and rock/concrete), *T*. *scripta* carapace (scute 3) and *T*. *scripta* plastron (scute 8) samples.
(PDF)

S5 Appendix Tables of ANCOM results. This file contains two tables: 1) “ANCOM Taxon Names”: a table of 16S and 18S taxa with significantly different distribution between environmental (wood and rock/concrete) and *T*. *scripta* carapace and plastron samples (scutes 3 and 8); 2) “Per-Sample Feature Counts”: a table of feature counts by sample for 16S and 18S taxa identified with significantly differing distributions between environmental (wood and rock/concrete) and *T*. *scripta* carapace and plastron samples (scutes 3 and 8), and for potentially pathogenic bacterial genera identified from our sequencing pools.
(XLSX)

## References

[1] Ross JR. Constraints on variables in syntax: Massachusetts Institute of Technology; 1967.

[2] Woodhams DC, Bletz MC, Becker CG, Bender HA, Buitrago-Rosas D, Diebboll H, et al Host-associated microbiomes are predicted by immune system complexity and climate. Genome Biol. 2020;21(1):23 10.1186/s13059-019-1908-8 32014020 PMC6996194

[3] Woodburn DB, Miller AN, Allender MC, Maddox CW, Terio KA. Emydomyces testavorans, a New Genus and Species of Onygenalean Fungus Isolated from Shell Lesions of Freshwater Aquatic Turtles. J Clin Microbiol. 2019;57(2). 10.1128/JCM.00628-18 30487306 PMC6355550

[4] Wang J, Yan M, Gao H, Lu X, Kan B. Vibrio cholerae Colonization of Soft-Shelled Turtles. Appl Environ Microbiol. 2017;83(14). 10.1128/AEM.00713-17 28600312 PMC5494633

[5] Cook DG, Martini-Lamb J. Distribution and habitat use of Pacific pond turtles in a summer impounded river. Transactions of the Western Section of the Wildlife Society. 2004;40:84–9.

[6] Fuselier L, Edds D. Habitat partitioning among three sympatric species of map turtles, genus Graptemys. Journal of Herpetology. 1994;28:154–8.

[7] Harrel JB, Allen CM, Hebert SJ. Movements and habitat use of subadult alligator snapping turtles (Macroclemys temminckii) in Louisiana. Am Midl Nat. 1996;135(1):60–7.

[8] Moore MJC, Seigel RA. No place to nest or bask: Effects of human disturbance on the nesting and basking habits of yellow-blotched map turtles (Graptemys flauimaculata). Biol Conserv. 2006;130(3):386–93.

[9] Pluto TG, Bellis ED. Habitat Utilization by the Turtle, Graptemys-Geographica, Along a River. Journal of Herpetology. 1986;20(1):22–31.

[10] Reese DA, Welsh HH. Habitat use by western pond turtles in the Trinity River, California. J Wildlife Manage. 1998;62(3):842–53.

[11] Herbert CV, Jackson DC. Temperature Effects on the Responses to Prolonged Submergence in the Turtle Chrysemys-Picta-Bellii .2. Metabolic-Rate, Blood Acid-Base and Ionic Changes, and Cardiovascular Function in Aerated and Anoxic Water. Physiol Zool. 1985;58(6):670–81.

[12] Frick MG, Williams KL, Markesteyn EJ, Pfaller JB, Frick RE. New records and observations of epibionts from loggerhead sea turtles. Southeast Nat. 2004;3(4):613–20.

[13] Majewska R, Santoro M, Bolanos F, Chaves G, De Stefano M. Diatoms and Other Epibionts Associated with Olive Ridley (Lepidochelys olivacea) Sea Turtles from the Pacific Coast of Costa Rica. Plos One. 2015;10(6). 10.1371/journal.pone.0130351 26083535 PMC4471233

[14] Majewska R, Van de Vijver B, Nasrolahi A, Ehsanpour M, Afkhami M, Bolanos F, et al Shared Epizoic Taxa and Differences in Diatom Community Structure Between Green Turtles (Chelonia mydas) from Distant Habitats. Microb Ecol. 2017;74(4):969–78. 10.1007/s00248-017-0987-x 28477173

[15] Robinson NJ, Majewska R, Lazo-Wasem EA, Nel R, Paladino FV, Rojas L, et al Epibiotic Diatoms Are Universally Present on All Sea Turtle Species. Plos One. 2016;11(6). 10.1371/journal.pone.0157011 27257972 PMC4892466

[16] MC P. Note on an alga (Dermatophyton radicans, Peter) growing on the European tortoise. Botanical Journal of the Linnaean Society. 1887;24(161):251–4.

[17] Hoffmann WE, Tilden JE. Basicladia, a new genus of Cladophoraceae. Bot Gaz. 1930;89:374–84.

[18] Proctor VW. The Growth of Basicladia on Turtles. Ecology. 1958;39(4):634–45.

[19] Gibbons J. Carapacial incidence of leech infestation in the painted turtle, Chrysemys picta. American Midlands Naturalist. 1968;79:517–9.

[20] Wu SC, Bergey EA. Diatoms on the carapace of common snapping turtles: Luticola spp. dominate despite spatial variation in assemblages. Plos One. 2017;12(2). 10.1371/journal.pone.0171910 28192469 PMC5305193

[21] Rivera SF, Vasselon V, Ballorain K, Carpentier A, Wetzel CE, Ector L, et al DNA metabarcoding and microscopic analyses of sea turtles biofilms: Complementary to understand turtle behavior. Plos One. 2018;13(4). 10.1371/journal.pone.0195770 29659610 PMC5901997

[22] Zimmerman J, Glockner G, Jahn R, Enke N, Gemeinholzer B. Metabarcoding vs. Morphological Identification to Assess Diatom Diversity in Environmental Studies. Molecular Ecology Resources. 2015;15:526–42. 10.1111/1755-0998.12336 25270047

[23] McKnight DT, Zenger KR, Alford RA, Huerlimann R. Microbiome diversity and composition varies across body areas in a freshwater turtle. Microbiology. 2020. 10.1099/mic.0.000904 32213245

[24] McKenzie VJ, Bowers RM, Fierer N, Knight R, Lauber CL. Co-habiting amphibian species harbor unique skin bacterial communities in wild populations. ISME J. 2012;6(3):588–96. 10.1038/ismej.2011.129 21955991 PMC3280140

[25] Rebollar EA, Hughey MC, Harris RN, Domangue RJ, Medina D, Ibanez R, et al The Lethal Fungus Batrachochytrium dendrobatidis Is Present in Lowland Tropical Forests of Far Eastern Panama. Plos One. 2014;9(4). 10.1371/journal.pone.0095484 24740162 PMC3989334

[26] Elbrecht V, Steinke D. Scaling up DNA metabarcoding for freshwater macrozoobenthos monitoring. Freshwater Biology. 2019;64(2):380–7.

[27] Takahashi S, Tomita J, Nishioka K, Hisada T, Nishijima M. Development of a Prokaryotic Universal Primer for Simultaneous Analysis of Bacteria and Archaea Using Next-Generation Sequencing. Plos One. 2014;9(8). 10.1371/journal.pone.0105592 25144201 PMC4140814

[28] Bradley IM, Pinto AJ, Guest JS. Design and Evaluation of Illumina MiSeq-Compatible, 18S rRNA Gene-Specific Primers for Improved Characterization of Mixed Phototrophic Communities. Appl Environ Microb. 2016;82(19):5878–91. 10.1128/AEM.01630-16 27451454 PMC5038042

[29] Tanabe AS, Nagai S, Hida K, Yasuike M, Fujiwara A, Nakamura Y, et al Comparative study of the validity of three regions of the 18S-rRNA gene for massively parallel sequencing-based monitoring of the planktonic eukaryote community. Molecular Ecology Resources. 2016;16(2):402–14. 10.1111/1755-0998.12459 26309223

[30] Martin M. Cutadapt removes adapter sequences from high-throughput sequencing reads. EMBnetjournal. 2011;17:10–2.

[31] Bolyen E, Rideout JR, Dillon MR, Bokulich NA, Abnet CC, Al-Ghalith GA, et al Reproducible, interactive, scalable and extensible microbiome data science using QIIME 2. Nat Biotechnol. 2019;37(8):852–7. 10.1038/s41587-019-0209-9 31341288 PMC7015180

[32] Callahan BJ, McMurdie PJ, Rosen MJ, Han AW, Johnson AJ, Holmes SP. DADA2: High-resolution sample inference from Illumina amplicon data. Nat Methods. 2016;13(7):581–3. 10.1038/nmeth.3869 27214047 PMC4927377

[33] Quast C, Pruesse E, Yilmaz P, Gerken J, Schweer T, Yarza P, et al The SILVA ribosomal RNA gene database project: improved data processing and web-based tools. Nucleic Acids Research. 2012;41(D1):D590–D6. 10.1093/nar/gks1219 23193283 PMC3531112

[34] Bates D, Machler M, Bolker B, Walker S. Fitting Linear Mixed-Effects Models Using lme4. Journal of Statistical Software. 2015;67(1):1–48.

[35] Luke SG. Evaluating significance in linear mixed-effects models in R. Behav Res Methods. 2017;49(4):1494–502. 10.3758/s13428-016-0809-y 27620283

[36] Oksanen J, Guillaume Blanchett F, Friendly M, Kindt R, Legendre P, McGlinn D, et al. vegan: Community Ecology Package. R package version 2.5–6 https://CRAN.R-project.org/package=vegan2019.

[37] Benjamini Y, Drai D, Elmer G, Kafkafi N, Golani I. Controlling the false discovery rate in behavior genetics research. Behav Brain Res. 2001;125(1–2):279–84. 10.1016/s0166-4328(01)00297-2 11682119

[38] Wickham H. ggplot2: Elegant Graphics for Data Analysis. New York: Springer-Verlag; 2016.

[39] McMurdie PJ, Holmes S. phyloseq: an R package for reproducible interactive analysis and graphics of microbiome census data. PLoS One. 2013;8(4):e61217 10.1371/journal.pone.0061217 23630581 PMC3632530

[40] Mandal S, van Treuren W, White R, Eggesbo M, Knight R, Peddada S. Analysis of Composition of Microbiomes: A Novel Method for Studying Microbial Composition. Microbial Ecology in Health and Disease. 2015;26:27663 10.3402/mehd.v26.27663 26028277 PMC4450248

[41] IUCN Red List 2019 [Available from: www.iucnredlist.org.

[42] Lovich J, Ennen J, Agha M, Gibbons J. Where Have All the Turtles Gone, and Why Does It Matter? Bioscience. 2018;68:771–8.

[43] Rhodin A, Stanford C, van Dijk P, Eisemberg C, Luiselli L, Mittermeier R, et al Global Conservation Status of Turtles and Tortoises (Order Testudines). Chelonian Conservation and Biology. 2018;17:135–61.

[44] Carpenter SR, Stanley EH, Zanden MJV. State of the World's Freshwater Ecosystems: Physical, Chemical, and Biological Changes. Annual Review of Environment and Resources. 2011;36(1):75–99.

[45] Rowe H, Whithey J, Neely M. Zebrafish as a model for zoonotic aquatic pathogens. Developmental and Comparative Immunology. 2014;46(1):96–107. 10.1016/j.dci.2014.02.014 24607289 PMC4096445

[46] Zeglin LH. Stream microbial diversity in response to environmental changes: review and synthesis of existing research. Frontiers in Microbiology. 2015;6 10.3389/fmicb.2015.00006 26042102 PMC4435045

[47] Sims A, Zhang Y, Gajaraj S, Brown PB, Hu Z. Toward the development of microbial indicators for wetland assessment. Water Res. 2013;47(5):1711–25. 10.1016/j.watres.2013.01.023 23384515

[48] Sievert G, Sievert L. A Field Guide to Oklahoma’s Amphibians and Reptiles: Oklahoma Department of Wildlife Conservation; 2005.

[49] Stone P, Powers S, Babb M. Freshwater Turtle Assemblages in Central Oklahoma Farm Ponds. The Southwestern Naturalist. 2005;50:166–71.

[50] Peterman W, William E, Travis J. Basking Behavior of Emydid Turtles (Chysemys picta, Graptemys geographica, and Trachemys scripta) in an Urban Landscape. Northeastern Naturalist. 2009;16(4):629–36.

[51] Becker K, Wahl M. Behaviour patterns as natural antifouling mechanisms of tropical marine crabs. J Exp Mar Biol Ecol. 1996;203(2):245–58.

[52] Wilson D, Tracy C, Tracy C. Estimating age of turtles from growth rings: a critical evaluation of the technique. Herpetologica. 2003;59(2):178–94.

[53] Krotman Y, Yergaliyev TM, Alexander Shani R, Avrahami Y, Szitenberg A. Dissecting the factors shaping fish skin microbiomes in a heterogeneous inland water system. Microbiome. 2020;8(1):9 10.1186/s40168-020-0784-5 32005134 PMC6995075

[54] Theodorakopoulos N, Bachar D, Christen R, Alain K, Chapon V. Exploration of Deinococcus-Thermus molecular diversity by novel group-specific PCR primers. Microbiologyopen. 2013;2(5):862–72. 10.1002/mbo3.119 23996915 PMC3831646

[55] Wang X, Liu X, Kono S, Wang G. The ecological perspective of microbial communities in two pairs of competitive Hawaiian native and invasive macroalgae. Microb Ecol. 2013;65(2):361–70. 10.1007/s00248-012-0144-5 23212654

[56] Robertson RR, Tezuka N, Watanabe MM. Phylogenetic analyses of Synechococcus strains (cyanobacteria) using sequences of 16S rDNA and part of the phycocyanin operon reveal multiple evolutionary lines and reflect phycobilin content. International Journal of Systematic and Evolutionary Microbiology. 2001;51:861–71. 10.1099/00207713-51-3-861 11411708

[57] Hubert W, Warner M. Control of *Epistylis* on channel catfish in raceways. Journal of Wildlife Diseases. 1975;11:241–4. 10.7589/0090-3558-11.2.241 806710

[58] Hudson D, Lester R. Parasites and symbionts of wild mud crabs Scylla serrata (Forskal) of potential significance in aquaculture. Aquaculture. 1994;120(3–4):183–99.

[59] Jones S, Carrasco NK, Vosloo A, Perissinotto R. Impacts of turbidity on an epibiotic ciliate in the St Lucia Estuary, South Africa. Hydrobiologia. 2018;815(1):37–46.

[60] Sladecek V. Indicator Value of the Genus Opercularia (Ciliata). Hydrobiologia. 1981;79(3):229–32.

[61] Taylor W. Rates of population increase and mortality for sessile ciliates on artificial substrates in a stream riffle. Canadian Journal of Zoology. 1983;61(9):2023–8.

[62] Verni F, Rosati G. Resting cysts: a survival strategy in Protozoa Ciliophora. Italian Journal of Zoology. 2011;78(2):134–45.

[63] Slugocki L, Karpowicz M, Kaczmarczyk-Ziemba A, Kozlowska J, Spikkeland I, Nilssen JP. Passenger for millenniums: association between stenothermic microcrustacean and suctorian epibiont—the case of Eurytemora lacustris and Tokophyra sp. Sci Rep. 2020;10(1):9577 10.1038/s41598-020-66730-2 32533081 PMC7293243

[64] Carda-Diéguez M, Ghai R, Rodríguez-Valera F, Amaro C. Wild eel microbiome reveals that skin mucus of fish could be a natural niche for aquatic mucosal pathogen evolution. Microbiome. 2017;5(1):162 10.1186/s40168-017-0376-1 29268781 PMC5740887

[65] Dalla Valle L, Nardi A, Toni M, Emera D, Alibardi L. Beta-keratins of turtle shell are glycine-proline-tyrosine rich proteins similar to those of crocodilians and birds. J Anat. 2009;214(2):284–300. 10.1111/j.1469-7580.2008.01030.x 19207990 PMC2667886

[66] Gibbons J, Greene J, Congdon J. Temporal and spatial movement patterns of sliders and other turtles In: Gibbons J, editor. Life History and Ecology of the Slider Turtle. Washington, D.C.: Smithsonian Institution; 1990 p. 201–15.

[67] Lowe S, Browne M, Boudjelas S, De Poorter M. 100 of the World’s Worst Invasive Alien Species: A Selection from the Global Invasive Species Database. The Invasive Species Specialist Group of the Species Survival Commission of the World Conservation Union2004.

