# Supplementary material for: Consistent patterns in 16S and 18S microbial diversity from the shells of the common and widespread red-eared slider turtle (Trachemys scripta)
Source: PLoS One. 2020 Dec 28;15(12):e0244489. doi: 10.1371/journal.pone.0244489 (PMC7769255; doi:10.1371/journal.pone.0244489)
Supplement: S1 Appendix — This file contains the indexing scheme applied in PCR1 amplifications and sequencing for turtle scute and environmental sample, and sequences of PCR primers used in 16S and 18S PCR1 and PCR2 amplifications. (PDF) [file pone.0244489.s001.pdf]

Indexing scheme for 96-well plate PCR amplifications.

Environmental and turtle samples names are indicated as:

Environmental: collection_date(YYYMMDD)-sample_number_for_date

Turtle: species-collection_date-turtle_number #scute_location

REVERSE<br>FORWARD	BR2_2<br>AA	BR2_3<br>TGG	BR2_4<br>GTAA	BR2_5<br>GAAA<br>G	BR2_6<br>TCCTG<br>A	BR2_B<br>GC	BR2_C<br>ATC	BR2_D<br>CACG	BR2_E<br>TCGTA	BR2_F<br>ACTAAT	BR2_X<br>CCAT	BR2_Z<br>CGGAG<br>A
BF2_2<br>**GA**	20190710-E1	20190713-E1	20190812-E2	TRSC-20190628-03 #1	TRSC-20190712-01 #1	TRSC-20190812-01 #1	TRSC-20190908-02 #1	PSCO-20190908-01 #1	TRSC-20190628-01 #3	TRSC-20190722-01 #3	TRSC-20190628-01 #8	TRSC-20190722-01 #8
BF2_T<br>**TGT**	20190710-E2	20190713-E2	20190901-E1	TRSC-20190628-03 #2	TRSC-20190712-01 #2	TRSC-20190812-01 #2	TRSC-20190908-02 #2	PSCO-20190908-01 #2	TRSC-20190628-02 #3	TRSC-20190722-02 #3	TRSC-20190628-02 #8	TRSC-20190722-02 #8
BF2_4<br>**CTTT**	20190702-E1	20190714-E1	20190901-E2	TRSC-20190628-03 #3	TRSC-20190712-01 #3	TRSC-20190812-01 #3	TRSC-20190908-02 #3	PSCO-20190908-01 #3	TRSC-20190702-01 #3	TRSC-20190812-02 #3	TRSC-20190702-01 #8	TRSC-20190812-02 #8
BF2_5<br>**TTTCA**	20190702-E2	20190714-E2	20190908-E1	TRSC-20190628-03 #4	TRSC-20190712-01 #4	TRSC-20190812-01 #4	TRSC-20190908-02 #4	PSCO-20190908-01 #4	TRSC-20190702-02 #3	TRSC-20190901-01 #3	TRSC-20190702-02 #8	TRSC-20190908-01 #8
BF2_6<br>**AAAGAA**	20190703-E1	20190722-E1	20190908-E2	TRSC-20190628-03 #5	TRSC-20190712-01 #5	TRSC-20190812-01 #5	TRSC-20190908-02 #5	PSCO-20190908-01 #5	TRSC-20190703-01 #3	TRSC-20190901-02 #3	TRSC-20190703-01 #8	TRSC-20190915-01 #8
BF2_B<br>**AT**	20190703-E2	20190722-E2	20190915-E1	TRSC-20190628-03 #6	TRSC-20190712-01 #6	TRSC-20190812-01 #6	TRSC-20190908-02 #6	PSCO-20190908-01 #6	TRSC-20190703-02 #3	TRSC-20190908-01 #3	TRSC-20190703-02 #8	TRSC-20190915-02 #8
BF2_C<br>**GTC**	20190712-E1	20190722-E3	20190915-E2	TRSC-20190628-03 #7	TRSC-20190712-01 #7	TRSC-20190812-01 #7	TRSC-20190908-02 #7	PSCO-20190908-01 #7	TRSC-20190713-01 #3	TRSC-20190915-01 #3	TRSC-20190713-01 #8	X
BF2_D<br>**AGAA**	20190712-E2	20190812-E1	TRSC-20190628-02 #7	TRSC-20190628-03 #8	TRSC-20190712-01 #8	TRSC-20190812-01 #8	TRSC-20190908-02 #8	PSCO-20190908-01 #8	TRSC-20190714-02 #3	TRSC-20190915-02 #3	TRSC-20190714-02 #8	X

Primer sequences (fasta formatted) used in 16S and 18S PCR1 and PCR2 amplifications.

PCR1:
16S forward primers:
>16s_Pro341F_P5_BF2_2
CACTCTTTCCCTACACGACGCTCTTCCGATCTGACCTACGGGNBGCASCAG
>16s_Pro341F_P5_BF2_T
CACTCTTTCCCTACACGACGCTCTTCCGATCTTGTCCTACGGGNBGCASCAG
>16s_Pro341F_P5_BF2_4
CACTCTTTCCCTACACGACGCTCTTCCGATCTCTTTCCTACGGGNBGCASCAG
>16s_Pro341F_P5_BF2_5
CACTCTTTCCCTACACGACGCTCTTCCGATCTTTTCACCTACGGGNBGCASCAG
>16s_Pro341F_P5_BF2_6
CACTCTTTCCCTACACGACGCTCTTCCGATCTAAAGAACCTACGGGNBGCASCAG
>16s_Pro341F_P5_BF2_B
CACTCTTTCCCTACACGACGCTCTTCCGATCTATCCTACGGGNBGCASCAG
>16s_Pro341F_P5_BF2_C
CACTCTTTCCCTACACGACGCTCTTCCGATCTGTCCCTACGGGNBGCASCAG
>16s_Pro341F_P5_BF2_D
CACTCTTTCCCTACACGACGCTCTTCCGATCTAGAACCTACGGGNBGCASCAG

16S reverse primers:
>16s_Pro805R_P7_BR2_2
TGACTGGAGTTCAGACGTGTGCTCTTCCGATCTAAGACTACNVGGGTATCTAATCC
>16s_Pro805R_P7_BR2_3
TGACTGGAGTTCAGACGTGTGCTCTTCCGATCTTGGGACTACNVGGGTATCTAATCC
>16s_Pro805R_P7_BR2_4
TGACTGGAGTTCAGACGTGTGCTCTTCCGATCTGTAAGACTACNVGGGTATCTAATCC
>16s_Pro805R_P7_BR2_5
TGACTGGAGTTCAGACGTGTGCTCTTCCGATCTGAAAGGACTACNVGGGTATCTAATCC
>16s_Pro805R_P7_BR2_6
TGACTGGAGTTCAGACGTGTGCTCTTCCGATCTTCCTGAGACTACNVGGGTATCTAATCC
>16s_Pro805R_P7_BR2_B
TGACTGGAGTTCAGACGTGTGCTCTTCCGATCTGCGACTACNVGGGTATCTAATCC
>16s_Pro805R_P7_BR2_C
TGACTGGAGTTCAGACGTGTGCTCTTCCGATCTATCGACTACNVGGGTATCTAATCC
>16s_Pro805R_P7_BR2_D
TGACTGGAGTTCAGACGTGTGCTCTTCCGATCTCACGGACTACNVGGGTATCTAATCC
>16s_Pro805R_P7_BR2_E
TGACTGGAGTTCAGACGTGTGCTCTTCCGATCTTCGTAGACTACNVGGGTATCTAATCC
>16s_Pro805R_P7_BR2_F
TGACTGGAGTTCAGACGTGTGCTCTTCCGATCTACTAATGACTACNVGGGTATCTAATCC
>16s_Pro805R_P7_BR2_X
TGACTGGAGTTCAGACGTGTGCTCTTCCGATCTCCATGACTACNVGGGTATCTAATCC

>16s_Pro805R_P7_BR2_Z
TGACTGGAGTTCAGACGTGTGCTCTTCCGATCTCGGAGAGACTACNVGGGTATCTAATCC

18S forward primers:
>18s_V8f_P5_BF2_2
CACTCTTTCCCTACACGACGCTCTTCCGATCTGAATAACAGGTCTGTGATGCCCT
>18s_V8f_P5_BF2_T
CACTCTTTCCCTACACGACGCTCTTCCGATCTTGTATAACAGGTCTGTGATGCCCT
>18s_V8f_P5_BF2_4
CACTCTTTCCCTACACGACGCTCTTCCGATCTCTTTATAACAGGTCTGTGATGCCCT
>18s_V8f_P5_BF2_5
CACTCTTTCCCTACACGACGCTCTTCCGATCTTTTCAATAACAGGTCTGTGATGCCCT
>18s_V8f_P5_BF2_6
CACTCTTTCCCTACACGACGCTCTTCCGATCTAAAGAAATAACAGGTCTGTGATGCCCT
>18s_V8f_P5_BF2_B
CACTCTTTCCCTACACGACGCTCTTCCGATCTATATAACAGGTCTGTGATGCCCT
>18s_V8f_P5_BF2_C
CACTCTTTCCCTACACGACGCTCTTCCGATCTGTCATAACAGGTCTGTGATGCCCT
>18s_V8f_P5_BF2_D
CACTCTTTCCCTACACGACGCTCTTCCGATCTAGAAATAACAGGTCTGTGATGCCCT

18S reverse primers:
>18s_1510r_P7_BR2_2
TGACTGGAGTTCAGACGTGTGCTCTTCCGATCTAACCTTCYGCAGGTTCACCTAC
>18s_1510r_P7_BR2_3
TGACTGGAGTTCAGACGTGTGCTCTTCCGATCTTGGCCTTCYGCAGGTTCACCTAC
>18s_1510r_P7_BR2_4
TGACTGGAGTTCAGACGTGTGCTCTTCCGATCTGTAACCTTCYGCAGGTTCACCTAC
>18s_1510r_P7_BR2_5
TGACTGGAGTTCAGACGTGTGCTCTTCCGATCTGAAAGCCTTCYGCAGGTTCACCTAC
>18s_1510r_P7_BR2_6
TGACTGGAGTTCAGACGTGTGCTCTTCCGATCTTCCTGACCTTCYGCAGGTTCACCTAC
>18s_1510r_P7_BR2_B
TGACTGGAGTTCAGACGTGTGCTCTTCCGATCTGCCCTTCYGCAGGTTCACCTAC
>18s_1510r_P7_BR2_C
TGACTGGAGTTCAGACGTGTGCTCTTCCGATCTATCCCTTCYGCAGGTTCACCTAC
>18s_1510r_P7_BR2_D
TGACTGGAGTTCAGACGTGTGCTCTTCCGATCTCACGCCTTCYGCAGGTTCACCTAC
>18s_1510r_P7_BR2_E
TGACTGGAGTTCAGACGTGTGCTCTTCCGATCTTCGTACCTTCYGCAGGTTCACCTAC
>18s_1510r_P7_BR2_F
TGACTGGAGTTCAGACGTGTGCTCTTCCGATCTACTAATCCTTCYGCAGGTTCACCTAC
>18s_1510r_P7_BR2_X

TGACTGGAGTTCAGACGTGTGCTCTTCCGATCTCCATCCTTCYGCAGGTTCACCTAC
>18s_1510r_P7_BR2_Z
TGACTGGAGTTCAGACGTGTGCTCTTCCGATCTCGGAGACCTTCYGCAGGTTCACCTAC

PCR2:
Universal forward primer:
>Univ_miseq_F
AATGATACGGCGACCACCGAGATCTACACTCTTTCCCTACACGACGCTC

Universal reverse primer:
>Univ_miseq_R
CAAGCAGAAGACGGCATACGAGATGTGACTGGAGTTCAGACGTGTGC
