# Supplementary material for: Consistent patterns in 16S and 18S microbial diversity from the shells of the common and widespread red-eared slider turtle (Trachemys scripta)
Source: PLoS One. 2020 Dec 28;15(12):e0244489. doi: 10.1371/journal.pone.0244489 (PMC7769255; doi:10.1371/journal.pone.0244489)

16S alpha rarefaction curves: Observed OTUs

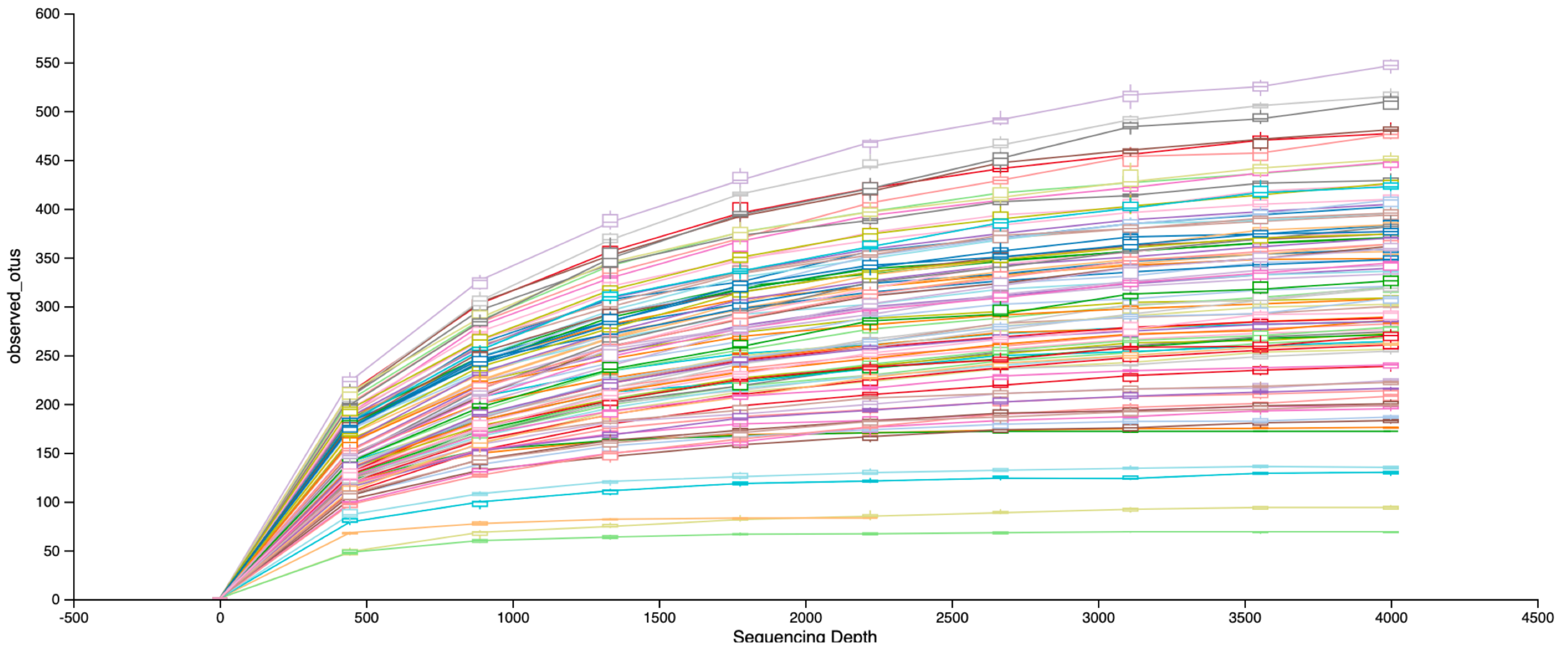

18S alpha rarefaction curves: Observed OTUs

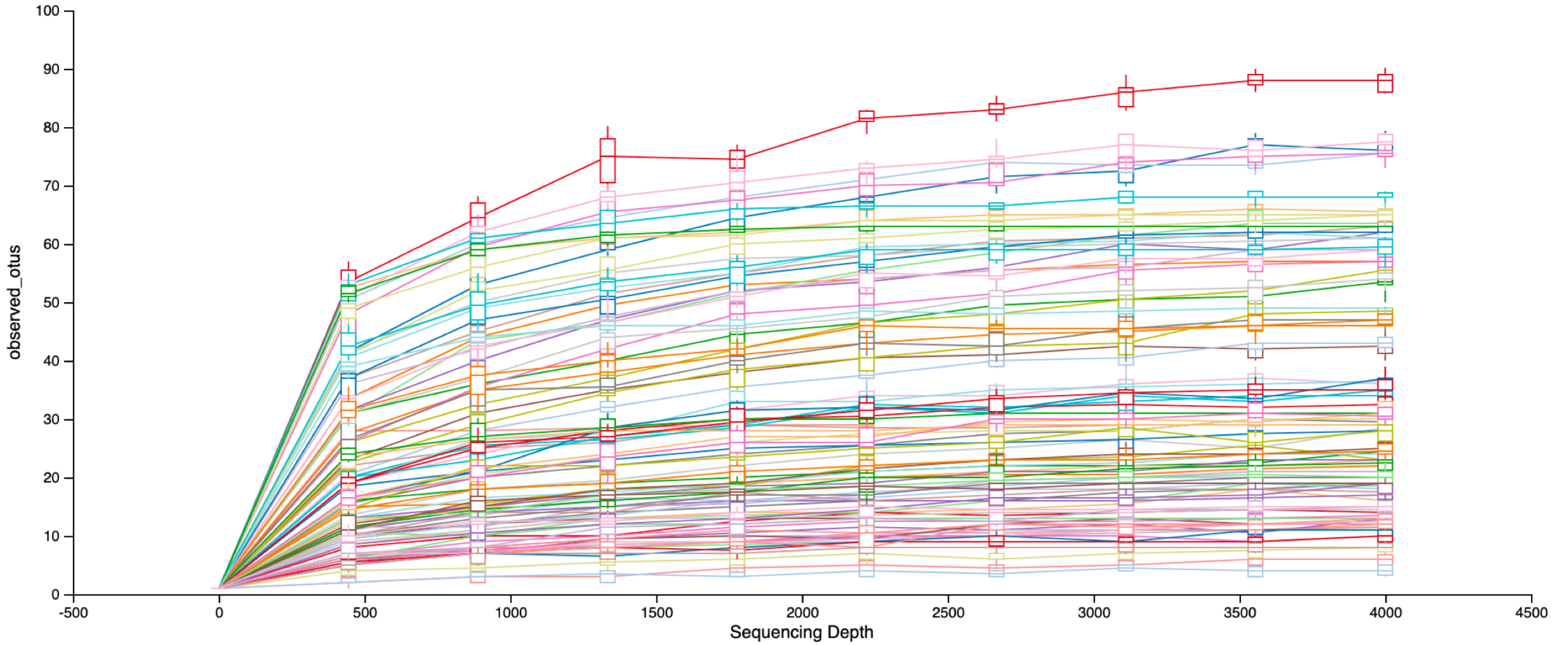

16S alpha rarefaction curves: Faith's Phylogenetic Diversity

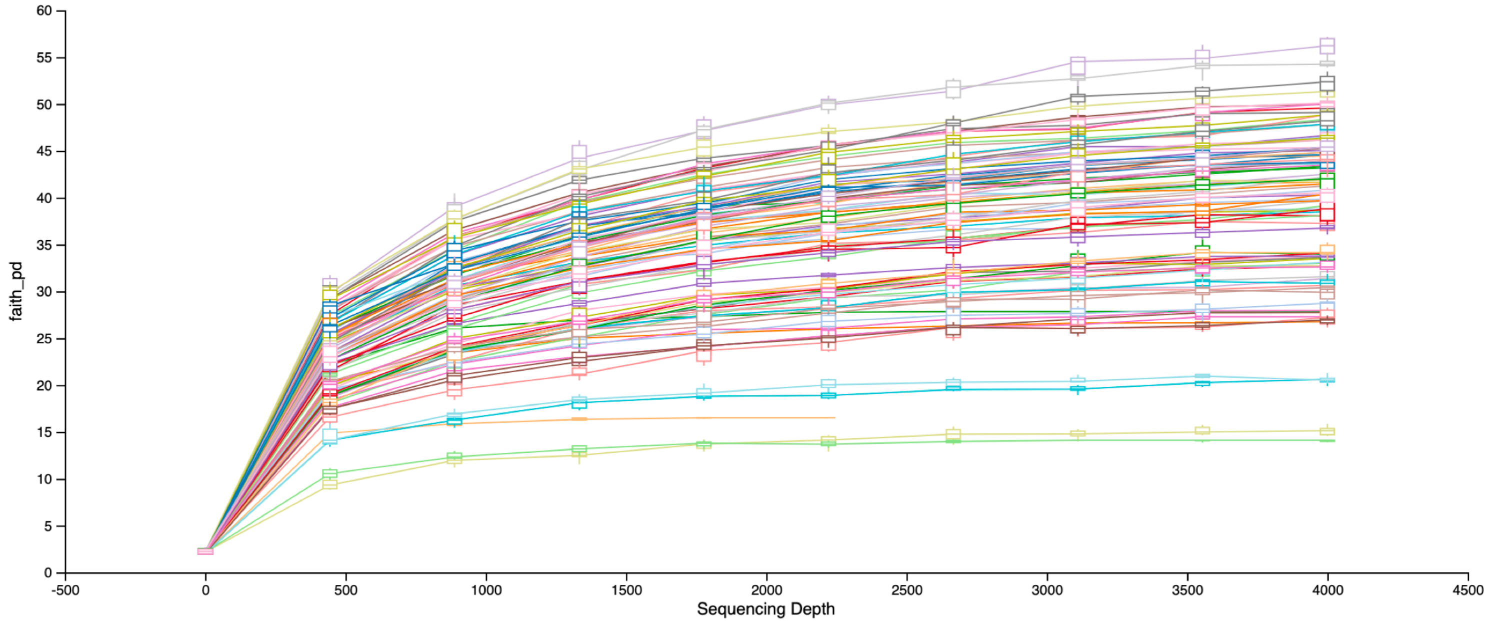

18S alpha rarefaction curves: Faith's Phylogenetic Diversity

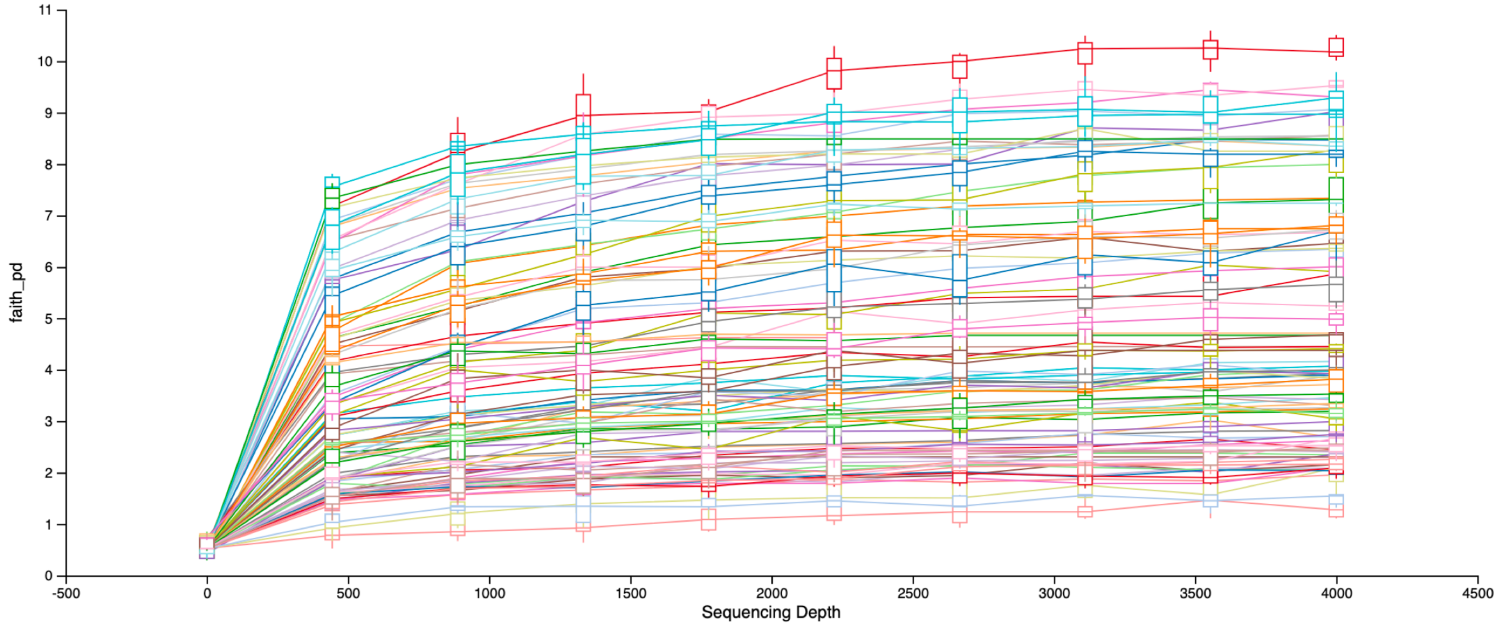

16S alpha rarefaction curves: Shannon Diversity

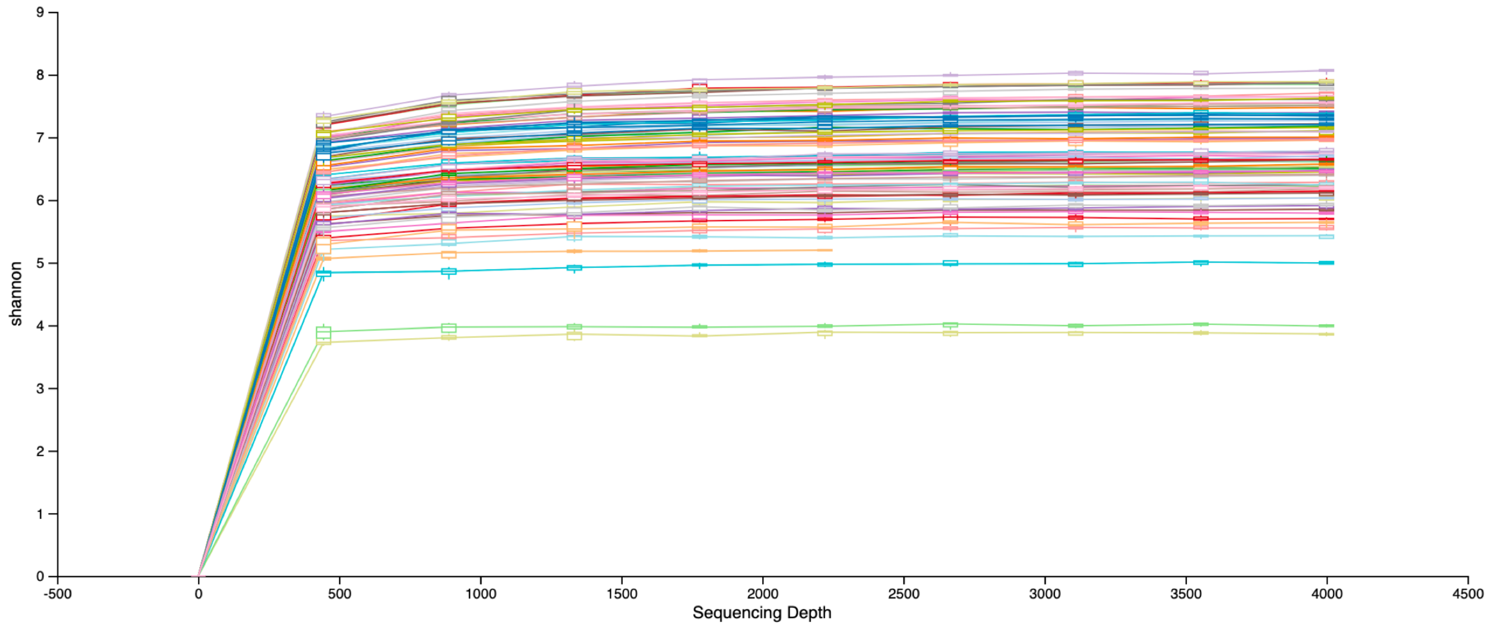

18S alpha rarefaction curves: Shannon Diversity

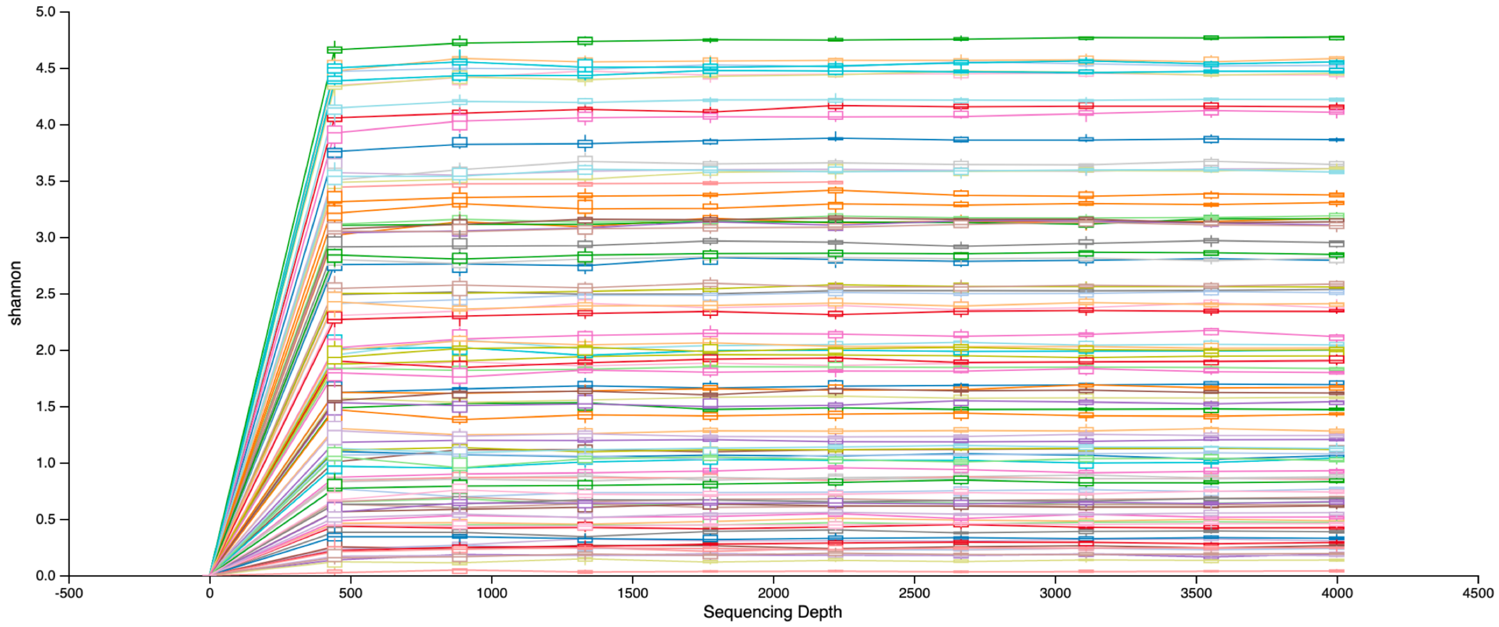

Supplement: S2 Appendix — This folder contains comma-delimited alpha rarefaction tables for 16 and 18S Faith’s Phylogenetic Diversity, Observed OTUs and Shannon diversity for all samples, as well as the corresponding alpha rarefaction curve images for these diversity indices for all samples. Identifying sample information is found in the final three columns of each rarefaction table. (ZIP) [file pone.0244489.s002.zip › S2_Appendix/alpha_rarefaction_curves.images.pdf]
