# Supplementary material for: Consistent patterns in 16S and 18S microbial diversity from the shells of the common and widespread red-eared slider turtle (Trachemys scripta)
Source: PLoS One. 2020 Dec 28;15(12):e0244489. doi: 10.1371/journal.pone.0244489 (PMC7769255; doi:10.1371/journal.pone.0244489)

4.1. Alpha diversity comparisons of environmental, carapace (*T. scripta* scute 3) and plastron (*T. scripta* scute 8) microbial communities. Column A) alpha diversity metrics for 16S communities; column B) alpha diversity metrics for 18S communities.

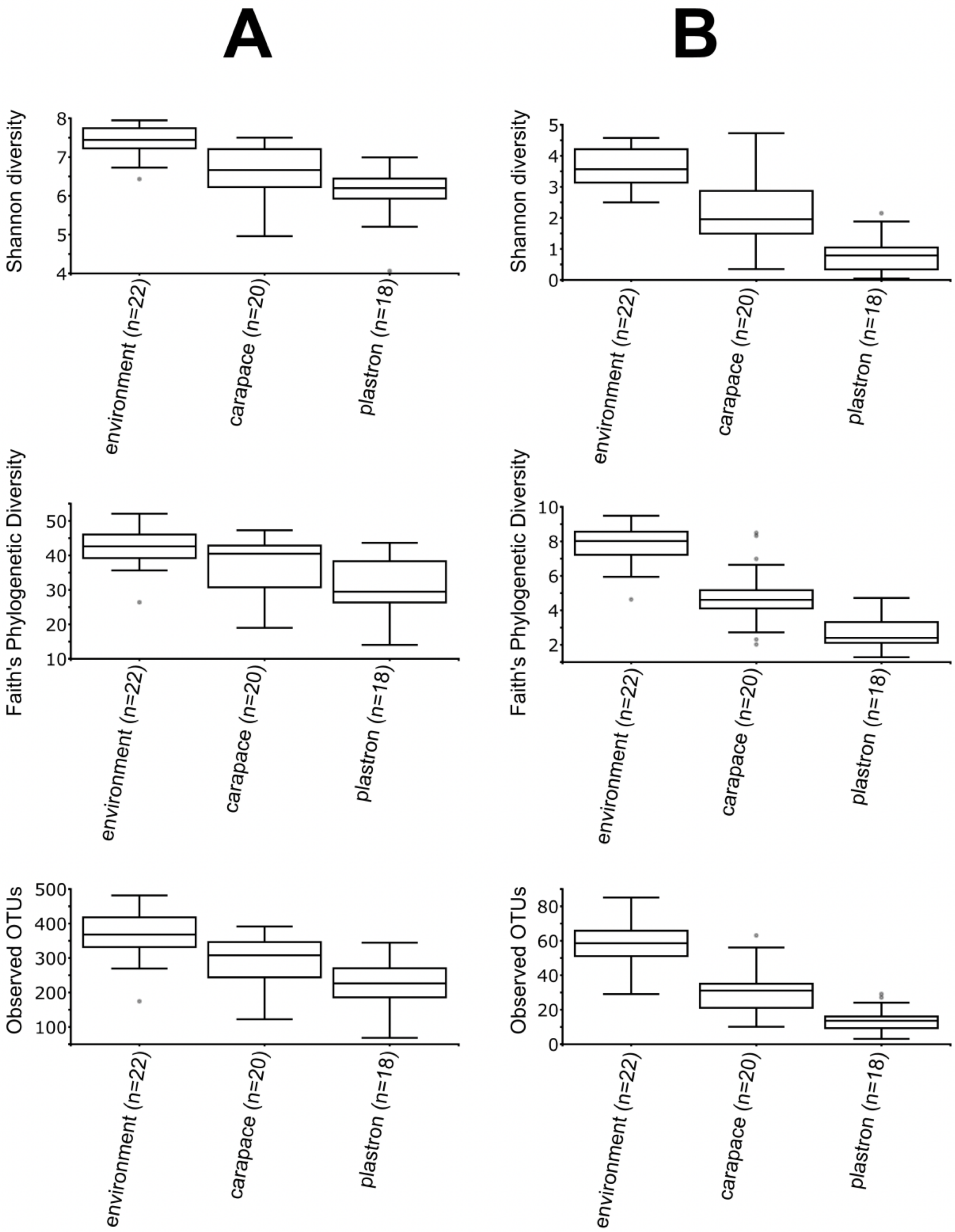

4.2. Beta diversity PCoA plots for all environmental and scute samples. For each row, left plot represents 16S communities; right plot represents 18S communities. Sample provenance indicated by shape and color (see legend). Row A) Bray-Curtis dissimilarity; row B) Jaccard dissimilarity; row C) unweighted UniFrac; row D) weighted UniFrac.

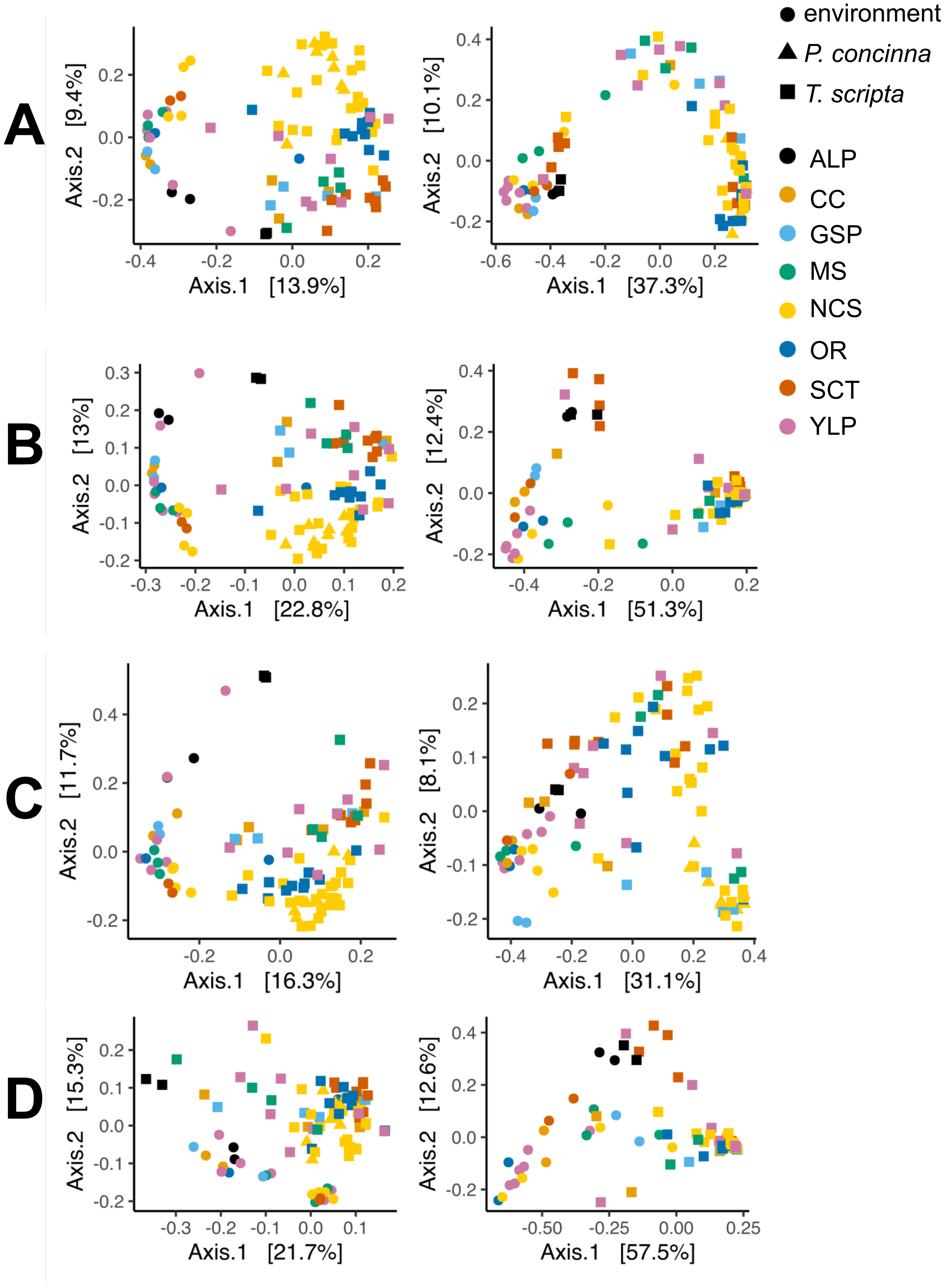

4.3. Beta diversity PCoA plots for environmental, *T. scripta* carapace (scute 3) and *T. scripta* plastron (scute 8) samples. For each row, left plot represents 16S communities; right plot represents 18S communities. Sample provenance indicated by color (see legend). Row A) Bray-Curtis dissimilarity; row B) Jaccard dissimilarity; row C) unweighted UniFrac; row D) weighted UniFrac.

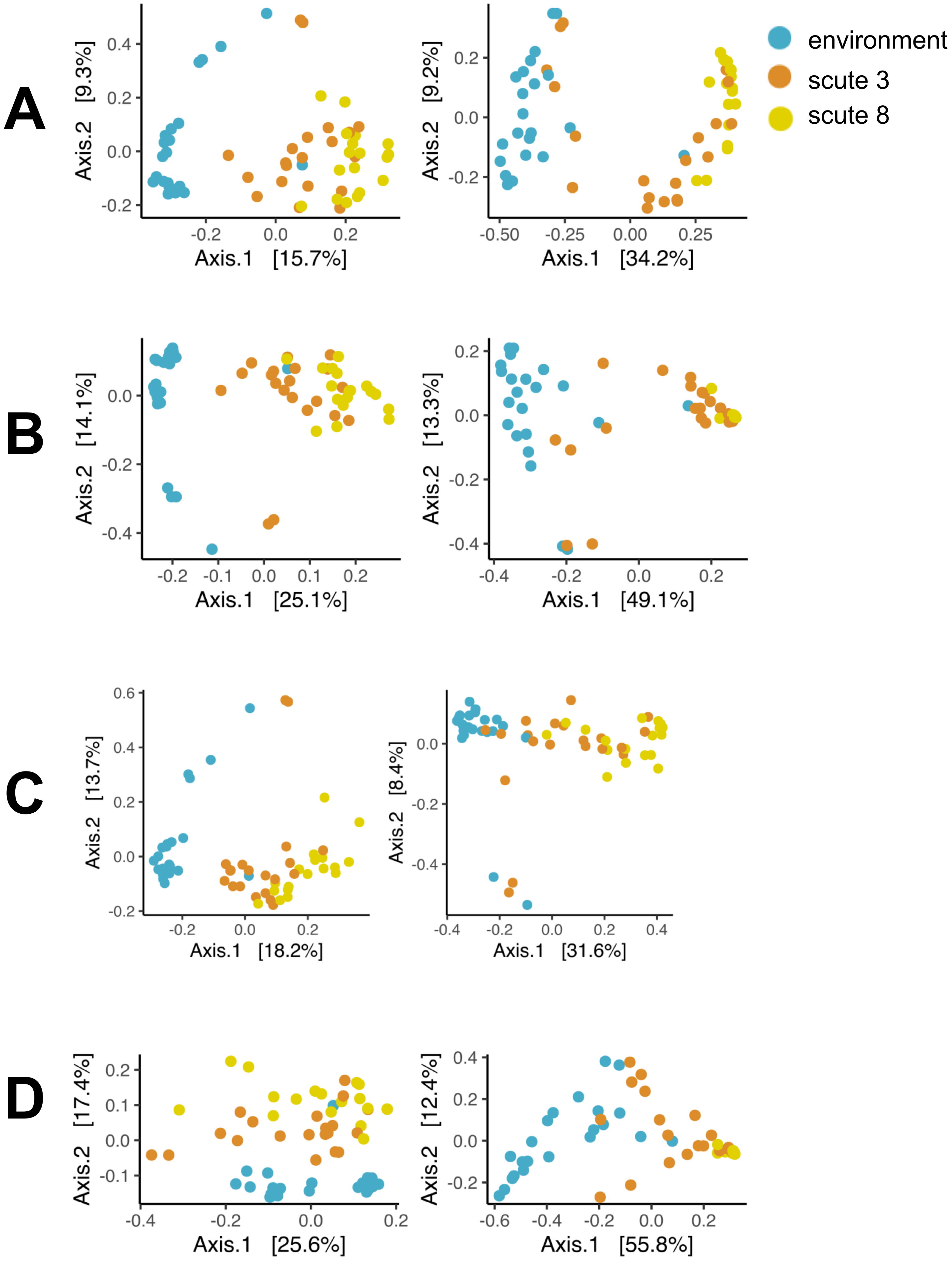

Supplement: S4 Appendix — This file contains: 4.1) alpha diversity box plots for all alpha diversity metrics applied, for 16S and 18S environmental (wood and rock/concrete), T. scripta carapace (scute 3) and T. scripta plastron (scute 8) comparisons; 4.2) Beta diversity emperor plots for all applied beta diversity metrics, for all 16S and 18S environmental and scute samples; 4.3) Beta diversity emperor plots for all applied beta diversity metrics, for 16S and 18S environmental (wood and rock/concrete), T. scripta carapace (scute 3) and T. scripta plastron (scute 8) samples. (PDF) [file pone.0244489.s004.pdf]
